# Regulation of cellular contractile force, shape and migration of fibroblasts by oncogenes and Histone deacetylase 6

**DOI:** 10.3389/fmolb.2023.1197814

**Published:** 2023-07-20

**Authors:** Ana López-Guajardo, Azeer Zafar, Khairat Al Hennawi, Valentina Rossi, Abdulaziz Alrwaili, Jessica D. Medcalf, Mark Dunning, Niklas Nordgren, Torbjörn Pettersson, Ian D. Estabrook, Rhoda J. Hawkins, Annica K. B. Gad

**Affiliations:** ^1^ Department of Oncology and Metabolism, The Medical School, University of Sheffield, Sheffield, United Kingdom; ^2^ Immunology and Molecular Oncology Diagnostics, Veneto Institute of Oncology IOV-IRCCS, Padova, Italy; ^3^ Bioinformatics Core, The Medical School, The University of Sheffield, Sheffield, United Kingdom; ^4^ Division Bioeconomy and Health, RISE Research Institutes of Sweden, Stockholm, Sweden; ^5^ Fibre and Polymer Technology, KTH Royal Institute of Technology, Stockholm, Sweden; ^6^ Department of Physics and Astronomy, University of Sheffield, Sheffield, United Kingdom; ^7^ Center for Advancing Electronics Dresden, Technische Universität Dresden, Dresden, Germany; ^8^ African Institute for Mathematical Sciences, Accra, Ghana; ^9^ Madeira Chemistry Research Centre, University of Madeira, Funchal, Portugal; ^10^ Department of Oncology-Pathology, Karolinska Institutet, Stockholm, Sweden

**Keywords:** Traction force microscopy, cellular contractile forces, intracellular forces on nucleus, fibroblasts, cell adhesion, Histone deacetylase 6, oncogenes, metastasis

## Abstract

The capacity of cells to adhere to, exert forces upon and migrate through their surrounding environment governs tissue regeneration and cancer metastasis. The role of the physical contractile forces that cells exert in this process, and the underlying molecular mechanisms are not fully understood. We, therefore, aimed to clarify if the extracellular forces that cells exert on their environment and/or the intracellular forces that deform the cell nucleus, and the link between these forces, are defective in transformed and invasive fibroblasts, and to indicate the underlying molecular mechanism of control. Confocal, Epifluorescence and Traction force microscopy, followed by computational analysis, showed an increased maximum contractile force that cells apply on their environment and a decreased intracellular force on the cell nucleus in the invasive fibroblasts, as compared to normal control cells. Loss of HDAC6 activity by tubacin-treatment and siRNA-mediated HDAC6 knockdown also reversed the reduced size and more circular shape and defective migration of the transformed and invasive cells to normal. However, only tubacin-mediated, and not siRNA knockdown reversed the increased force of the invasive cells on their surrounding environment to normal, with no effects on nuclear forces. We observed that the forces on the environment and the nucleus were weakly positively correlated, with the exception of HDAC6 siRNA-treated cells, in which the correlation was weakly negative. The transformed and invasive fibroblasts showed an increased number and smaller cell-matrix adhesions than control, and neither tubacin-treatment, nor HDAC6 knockdown reversed this phenotype to normal, but instead increased it further. This highlights the possibility that the control of contractile force requires separate functions of HDAC6, than the control of cell adhesions, spreading and shape. These data are consistent with the possibility that defective force-transduction from the extracellular environment to the nucleus contributes to metastasis, via a mechanism that depends upon HDAC6. To our knowledge, our findings present the first correlation between the cellular forces that deforms the surrounding environment and the nucleus in fibroblasts, and it expands our understanding of how cells generate contractile forces that contribute to cell invasion and metastasis.

## 1 Introduction

The capacity of single cells to adhere to, change shape and to exert physical forces on the surrounding microenvironment governs the architecture and mechanical properties of tissues. Cell adhesion, shape and physical forces also control cell motility and invasion into the surrounding environment (Kraning-Rush et al., 2012). These cell properties thereby govern fundamental processes in health and disease, such as embryonic development, tissue regeneration such as wound healing, as well as the primary cause of patient death in cancer, cell invasion and metastasis. While the molecular mechanisms of cell adhesion have been studied in detail since the 1970s (Horwitz, 2012; Clevers, 2016), the capacity of single cells to exert physical forces, and the underlying molecular mechanisms remain to a large extent unknown.

In pioneering work, the group of Yu-li Wang described the forces that fibroblasts exert on the environment on a subcellular level and showed that the migration of fibroblasts on a 2D surface are generated at the leading edge of the cell by small nascent cell-matrix adhesions (Munevar et al., 2001a; Karen et al., 2001; Trichet et al., 2012). The cytoskeleton, and the cytoskeletal regulators Rho GTPases are key molecules in the generation of traction forces (Gad et al., 2012). In particular, RhoA-ROCK- activation of actomyosin-based contractility generates cellular contractile force (Burridge, 1996; Parsons et al., 2010; Feld et al., 2020). In contrast, the vimentin intermediate filaments have been shown to suppress contractile forces of the cells (Jiu et al., Kim et al., 2022). The microtubules also exert an indirect control of the acto-myosin generated forces, by the release of the RhoA activator GEF-H1 (Ren et al., 1998; Krendel et al., 2002; Binker et al., 2007; Rafiq et al., 2019; Deb Roy et al., 2022). The observation that the post-translational acetylation of microtubule causes release of GEF-H1 from microtubules, suggests that the microtubule acetylation can regulate contractile forces (Holenstein et al., 2019; Seetharaman et al., 2022). Microtubule acetylation is controlled by the cytoplasmic deacetylase Histone deacetylase 6 (HDAC6). HDAC6 promotes oncogene-induced cell transformation, cell motility and metastasis (Lee et al., 2008; Lafarga et al., 2012; Bance et al., 2019). For example, it is required for the proliferation and metastasis of melanoma cells (Stivarou et al., 2016; Hu et al., 2023). HDAC6 also stimulate the motility of cells, which can be due to the capacity of HDAC6 to deacetylate cytoplasmic non-histone targets, primarily of 𝛂-tubulin (Zilberman et al., 2009; Li et al., 2014).

The forces that cells exert on intracellular organelles, such as the nucleus are studied to a lesser extent than the forces cells exert on their microenvironment. However, it is known that the shape and the mechanical properties of the nucleus of the cell is governed by the cytoplasmic cytoskeletal forces, the composition and ratio of lamin A/C within the nuclear lamina, and the organisation of chromatin (Schape et al., 2009; Jain et al., 2013; Swift et al., 2013; Chiotaki et al., 2014; Schreiner et al., 2015; Dos Santos et al., 2021; Katiyar et al., 2022). The LINC protein complex physically connects the nuclear envelope to the cytoskeleton, and the cytoskeleton subjects the nucleus to compressive forces that deform the nucleus by the microtubules, F-actin stress fibres and vimentin intermediate filaments (Jiu et al., 2017; Alisafaei et al., 2019; Terriac et al., 2019; Dos Santos and Toseland, 2021). Actomyosin-based contractility can therefore exert both a direct and indirect, microtubule-mediated physical control of the shape and the morphology of the nucleus.

We have previously observed that expression of various oncogenes in primary, healthy dermal fibroblasts reduces the polymerisation of microtubules, actin, and vimentin filaments, results in thinner stress fibres and collapses the organisation of vimentin filaments in fibroblast cells (Jiu, 2018). These oncogene-induced effects were accompanied by increased cell invasion and mechanical stiffness of the cells and were mediated by HDAC6 (Ghassemi et al., 2012; Rathje et al., 2014; Jiu et al., 2015). We have also shown that oncogene-expressing and transformed and invasive fibroblasts show increased cell migration speed, a less directed cell migration, an increased inverse correlation between cell migration speed and directionality, loss of the elongated fibroblast cells shape. These changes that were reversed to normal upon inhibition of the tubulin-deacetylase HDAC6 with the specific inhibitor tubacin (Haggarty et al., 2003b; Haggarty et al., 2003; Ling et al., 2018; Evans et al., 2022). Given the importance of physical forces for cell motility, we therefore hypothesised that oncogenes and HDAC6 also regulate the physical traction forces that cells exert on their surrounding environment and on their nuclei.

In this present study, we aim to determine if transformed and invasive fibroblasts generate defective traction forces on the surrounding environment and/or on the cell nucleus, and to identify underlying molecular mechanisms of control. Our findings suggest that oncogenic transformation and invasion of cells is accompanied by an increased physical force that cells exert on their environment, combined with a decreased intracellular cytoplasmic force on the cell nucleus, by a mechanism that requires HDAC6. The data is consistent with the hypothesis that oncogenes, via the separate deacetylase-, and protein-binding functions of HDAC6, regulate physical forces, cell-matrix adhesions, spreading, shape, migration of cells, which promote the motility and invasion of cells in cancer.

## 2 Materials and methods

### 2.1 Cell culture and treatments

We used the BjhTERT-SV40T-H-RasV12 cells, and the isogenically matched immortalised BjhTERT cells as normal non-transformed and non-invasive control cells (Hahn et al., 1999). Cells were cultured in Dulbecco’s modified Eagle’s medium supplemented with 10% foetal bovine serum and 100 U/mL penicillin and 100 μg/mL streptomycin. These cell variants have been described previously, and can be considered as an isogenically matched model system of the transformation of primary cells to transformed and invasive cells, and they have been characterised previously with regard to their total gene transcription, their protein expression, as well as the cytoskeletal organisation, cell-matrix adhesion, and migration (Danielsson et al., 2013; Rönnlund et al., 2013; Rathje et al., 2014; Evans et al., 2022). The expression of the SV40LT and H-Ras G12V oncogenes in the human primary BJ-fibroblasts have previously been shown to create tumours that metastasise into kidney and lung tissue, as well as into the parenchyma (Sun et al., 2005). Cells were treated with 10 µM tubacin or DMSO.

### 2.2 siRNA transfection

Cells were seeded at 40,000 cells/well in P12 well plate (CC7682-7512, STARLAB International GmbH, Hamburg, Germany) incubated for 24 h in Dulbecco’s modified Eagle’s medium supplemented with 10% foetal bovine serum and 100 U/mL penicillin and 100 μg/mL streptomycin. Transfection was performed using HiPerFect Transfection Reagent (301704, Qiagen, Hilden, Germany) following manufacturer instructions. SV40TRasV12 cells were transfected with a combination of the Hs_HDAC6_5 and the Hs_HDAC6_6 FlexiTube siRNA (1027417, Qiagen, Hilden, Germany) or with negative control siRNA (1022076**,** Qiagen, Hilden, Germany) at a final concentration of 25 nM, and incubated 96 h prior to analysis.

### 2.3 Western blot

Total cellular protein extracts (25 ug/lane) were separated using precast Stain Free polyacrylamide gels (4%–20%) (4568094, Bio-Rad Laboratories Ltd.), and Western blot procedures were performed as described previously (Evans et al., 2022). The list of antibodies used is as follows: anti-mouse-acetylated-α-tubulin (T6793, Sigma Aldrich) (1:2,000), anti-rabbit-α-tubulin (ab176560, Abcam) (1:2,000), anti-mouse-GAPDH (60004, Proteintech) (1:2,000), and anti-rabbit-HDAC6 (NB100-56343, Novis Biologicals) (1:2,000). HRP-conjugated goat anti-mouse/rabbit secondary antibodies (GtxRb-004-DHRPX/GtxRb-003-DHRPX, Immunoreagents) (1:10,000). ChemiDoc MP Imaging System (Catalog: 12003154) and software (Image Lab Touch 2.4, version 1709691) were used for the quantification of the bands from the western blots. For calculations of the ratio between acetylated-alpha-tubulin/total alpha-tubulin, blots were stripped according to the manufacturer’s protocol, using a Stripping buffer (Cat: 21059, Thermoscientific), and confirmed the loss of signal prior to re-probing. The antibodies are commercially available, and the source files of the Western blots are shown in Supplementary Figure S1.

### 2.4 Immunofluorescence staining

The cells were fixed and permeabilized in PBS containing 3.7% formaldehyde, 0.2% Triton-X100, and stained as described previously (Gad et al., 2012), using anti-rat-vimentin (MAB2105, R&D systems) (1:100), anti-mouse-p-Tyr (sc-7020, Santa Cruz Biotechnology) (1:100), Goat anti-rat-Alexa-Fluor-647 (Invitrogen) (1:400), Goat anti-mouse-Alexa-Fluor 555 (Invitrogen) (1:400), Alexa-Fluor-488 phalloidin (Invitrogen) (1:400), DAPI (1:1,000). The cells were then imaged with a ZEISS LSM 980 confocal microscope, using the Zen software (Carl Zeiss Microscopy, Thornwood, United States).

### 2.5 Traction force microscopy

Traction force microscopy experiments were performed as previously described (Kim et al., 2022) using the collagen coated 12 kPa hydrogels with 0.2 μm Yellow/green fluorospheres MatrigenSoftTrac™ plates (Cell guidance-Cambridge, Matrigen, LLC, California United States). Cells were seeded in complete, serum-containg media, at 2,500 cells/cm^2^ and allowed to adhere overnight. Prior to imaging, the media was replaced by 1 mL of 0.05 μg/mL Hoechst (H3570; Invitrogen, Waltham, MA, United States) in complete media for 30 min, which thereafter was removed and replaced by 500 μL of complete media. Images of the fluorescent embedded beads and the cells were obtained using parallel imaging and a × 40 magnification on a ZeissCelldiscoverer7 Wide field Fluorescence Microscope (Carl Zeiss Microscopy, Thornwood, NY). The cells were thereafter detached by addition of 1 mL of 0.5% (w/v) Triton-X-100, 20 mM NH_4_OH in PBS, followed by imaging of the exact same location of the gel after 5 min. This buffer has previously been used to detach cells for Traction force microscopy (Franco-Barraza et al., 2016; Teo et al., 2020). Visual confirmation of cell detachment was thereafter obtained by gently moving the microscope stage. Displacement of beads before and after the removal of cells was tracked by particle imaging velocimetry followed by Fourier transform traction cytometry to estimate the corresponding cell traction force field, using a modified ImageJ macro kindly shared by Lee and Kumar, (2020) from a previously described method (Martiel et al., 2015). The total traction forces (in N) were measured by integrating traction forces over the cell area. The elastic energy (in J) stored in the gel to produce the observed deformation was calculated by summing the products of displacement with the force over the cell.

### 2.6 Migration assay

Cells were seeded at 1,000 cells/well in slide chambers (543079; Greiner Bio-One Frickenhausen, Germany) and incubated for 24 h. The medium was replaced with a fresh medium containing 0.05 μg/mL Hoechst (H3570; Invitrogen, Waltham, MA, United States) for 1 h before live-cell imaging. This was followed by 17 h live-cell imaging on Cell Discoverer 7 (Zeiss, Oberkochen, Germany), with images taken every 20 min.

### 2.7 Image analysis

The live-cell imaging videos were analysed with regard to the mean cell migration speed and persistence using TrackMate plugin by FIJI version 1.53, as previously described (Kim et al., 2022). For cell shape analysis, the images taken after 8.5 h were used, and individual cells were contoured by the freehand selection tool followed by quantification by shape descriptors by FIJI version 1.53, as described previously (Kim et al., 2022).

### 2.8 Nuclear force analysis

We calculated the force field required to produce the nuclear deformation observed between the attached and detached states of the cells using a previously described method (Estabrook et al., 2021). Briefly, the algorithm determines the deformation of a thin elastic shell, using an elastic energy minimisation Monte Carlo scheme to obtain the deformation field from only two outlines of the nucleus. We assume the following elastic parameters for the nuclei in all cases; Young’s Modulus E = 5 kPa and Poisson’s ratio *ν* = 0.5, presuming the elastic shell is an incompressible material, are used to represent the nucleus (Guilak et al., 2000; Caille et al., 2002; Lammerding, 2011). The nucleus image outlines are obtained within ImageJ. The algorithm was adapted to analyse the nuclear Hoechst images obtained from the attached and detached cells during traction force microscopy experiments, as described above. Total force was calculated from output files by summing the modulus of the force vectors over the perimeter of the cell. The deformations were quantified by defining a deformation index equal to the mean of the modulus of the deformation vectors divided by the perimeter of the undeformed shape. The R scripts to calculate the total force and the deformation are included in the Supplementary information, as Supplementary Texts S1, S2, respectively.

### 2.9 Colloidal probe force-mode atomic force microscopy

Atomic force microscopy (AFM) was used to measure force interaction with the aid of a colloidal probe in the form of indentations over the cell nuclei, as described earlier (Rathje et al., 2014). The force measurement data were converted into force curves and elasticity was calculated from the force curves by fitting a contact mechanic model to the interacting force on approach (Carl and Schillers, 2008). We analysed the cell stiffness (10 cells of each type) at applied normal forces in the range of 10–40 nN. The differences observed between the samples followed the same ranking without any statistically significant differences at the different applied forces.

### 2.10 Statistical analysis

The statistical analysis was performed in GraphPad Prism version 9.1.0 (GraphPad Prism Software, San Diego, CA, United States). For the migration, focal adhesion area, nuclear force and traction force microscopy analyses, ordinary one-way ANOVA statistical tests were used to compare the experimental groups for mean speed and linearity and shape, focal adhesion areas, nuclear force, energy, total and maximum cell force statistical multiple comparison, respectively. For the migration analysis, the statistical analysis was performed in GraphPad Prism version 9.1.0 (GraphPad Prism Software, San Diego, CA, United States). Ordinary one-way ANOVA statistical tests were used to compare the experimental groups for mean speed and linearity and shape statistical multiple comparison. *t*-test statistical tests were used to compare the contractile forces for traction force microscopy analysis of siRNA-treated cells. Outliers identification was performed using the ROUT method (Q = 1%).

## 3 Results

### 3.1 Tubacin-mediated inhibition of HDAC6 decreases the maximum traction force of invasive cells

**FIGURE 1:**
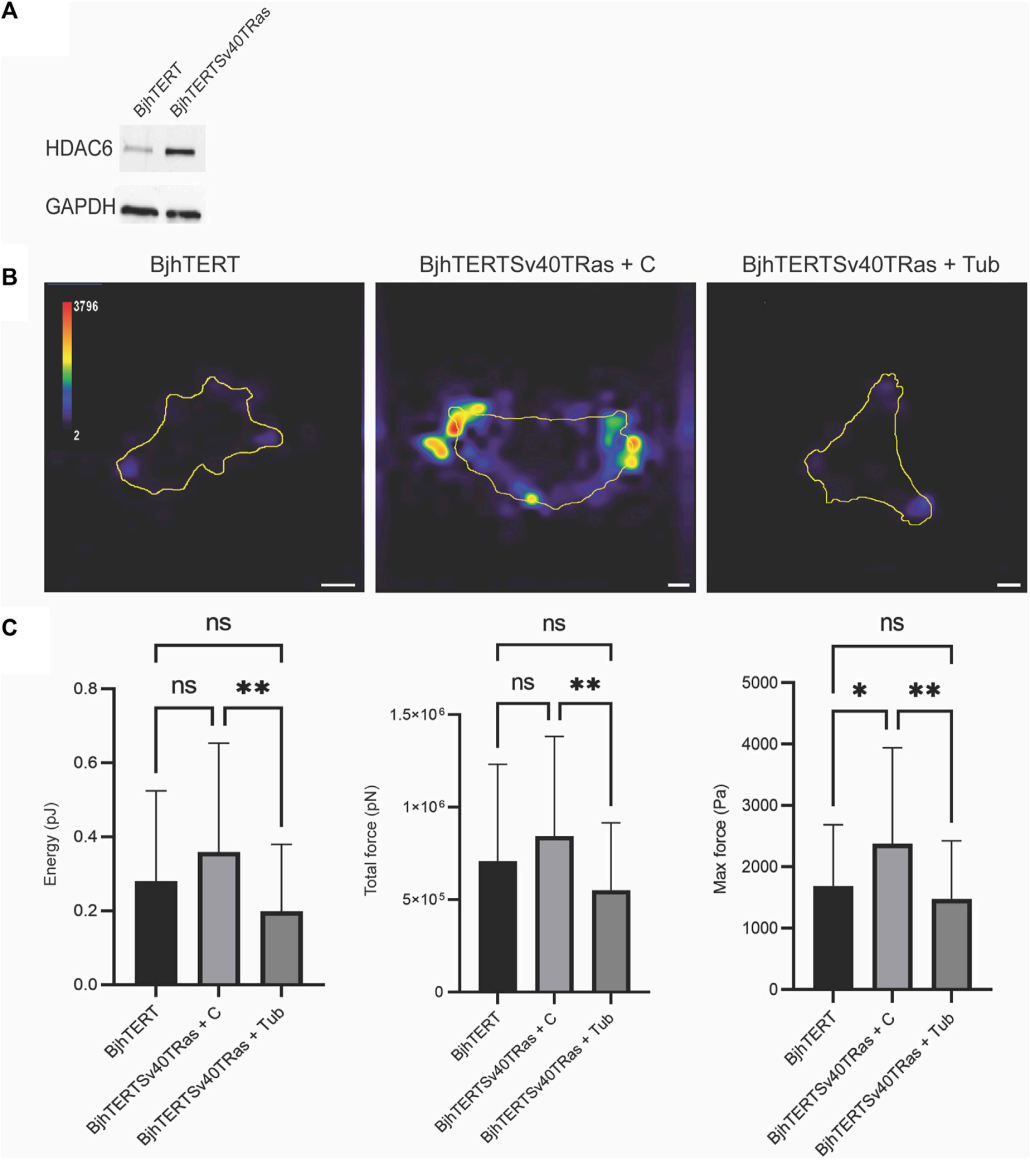
Transformed and invasive fibroblasts show increased maximum force, which is reversed to normal by tubacin-mediated inhibition of HDAC6. **(A)** The protein l evels of HDAC6 and GAPDH loading control in normal BjhTERT and metastasising BjhTERTSv40TRas cells, as indicated. **(B)** Representative traction force maps of control and Transformed and invasive fibroblasts, treated without or with tubacin (Tub), as indicated, with colour key indicating the magnitude of traction force, in Pascals. **(C)** The corresponding quantification graphs of the total strain energy (left), the total traction forces exerted by the cells (i.e., the traction forces integrated over cell area) (middle), and the maximum force of cells (right) are shown, as indicated. Data from at least three independent biological repeats, represented as mean ± SD. *p ≤ 0.05, **p ≤ 0.01 (One way ANOVA tests) (*t*-tests). For Energy and Total force analysis: BjhTERT n = 55, BjhTERTSv40TRas + C *n* = 54, BjhTERTSv40TRas + Tub *n* = 61. For Max Force analysis: BjhTERT *n* = 37, BjhTERTSv40TRas + C *n* = 49, BjhTERTSv40TRas + Tub *n* = 51. Scale bar: 10 µm.

Recent research has shown that the force that epithelial cells exert on their surrounding environment show a positive correlation to their capacity to invade (Kraning-Rush et al., 2012). To determine if this is the case also in mesenchymal cells, we therefore aimed to determine if oncogene-expressing, invasive and transformed and invasive fibroblasts show increased cellular contractile force, as compared to isogenically matched normal control cells. A main advantage with this cell model is that it allows direct comparison between cancer-forming human cells and isogenically matched control cells. As cancer is a systemic disease, it is not possible to obtain isogenically matched normal control cells to cancer cells or cell lines that have been derived from patients. Earlier observations by our and other laboratories have shown that oncogenes induce HDAC6 in fibroblasts (Rathje et al., 2014; Lea and Kumar, 2020). Accordingly, we observed increased levels of HDAC6 in the transformed and invasive fibroblasts (Figure 1). We therefore wished to determine if increased forces in transformed cells can depend upon HDAC6. Using traction force microscopy, we observed that the transformed and invasive cells showed similar levels of strain energy and traction forces as the control cells, while the level of the maximum contractile force in the cells was increased (*p* ≤ 0.01). Treatment with tubacin reduced the strain energy and the traction force, and also reduced the maximum force of transformed and invasive cells to normal (*p* ≤ 0.01, Figure 1). Taken together, these observations indicate that the capacity to exert contractile forces of the surrounding environment is increased in metastasising fibroblasts, possibly mediated by increased levels and/or activity of HDAC6.

### 3.2 siRNA-mediated HDAC6 knockdown increases the traction forces of invasive cells

**FIGURE 2:**
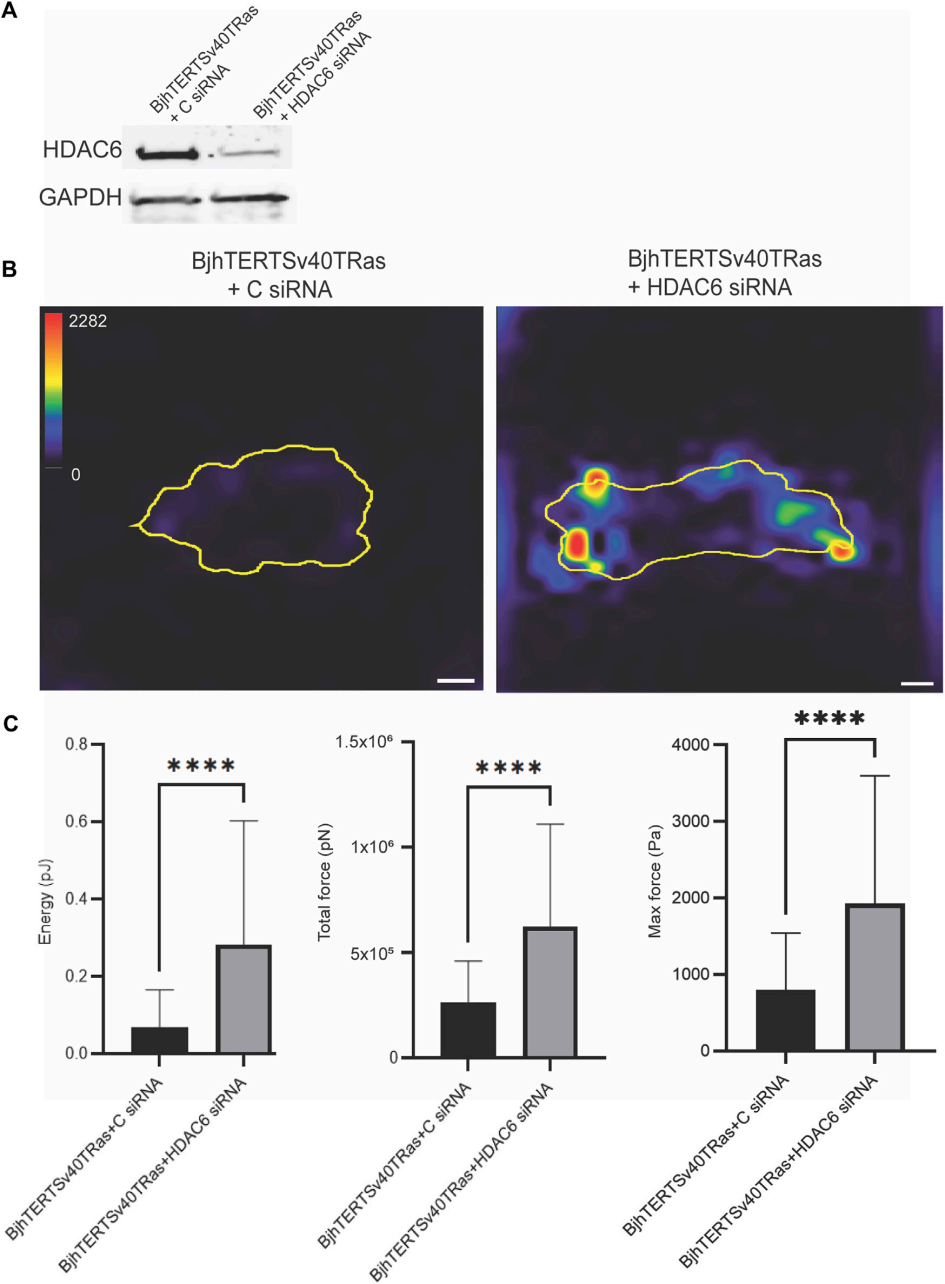
siRNA-mediated knockdown of HDAC6 increases the contractile forces in invasive cells. **(A)** The protein levels of HDAC6 and GAPDH loading control in cells transfected with HDAC6-targeting siRNA or control siRNA, as indicated. **(B)** Representative traction force maps of control and transformed and invasive fibroblasts, treated without or with HDAC6 siRNA as indicated with the colour key indicating the magnitude of traction force, in Pascals. The corresponding graphs of the total strain energy (left), the total traction forces (i.e., the traction forces integrated over the cell area) (middle), and the maximum force of cells (right) are shown, as indicated. Data from at least three independent biological repeats. Data presented as mean **p ≤ 0.01.± SD. *p ≤ 0.05 (One way ANOVA tests) (*t*-tests). BjhTERTSv40TRas + **(C)** siRNA *n* = 72, BjhTERTSv40TRas + HDAC6 siRNA *n* = 96. Scale bar: 10 µm.

We further wished to determine if not only the HDAC6 activity, but also the increased levels of the HDAC6 protein that we observed in the transformed and invasive cells were required for the increased contractile force of these cells. To this end, we knocked down HDAC6 with siRNA, and analysed the contractile forces in transformed and Invasive with or without HDAC6. We observed that loss of HDAC6 resulted in increased levels of strain energy (*p* ≤ 0.00011), traction force (*p* ≤ 0.00011) and maximum force (*p* ≤ 0.00011) in cells, as compared to control (Figure 2). These results suggest that the protein levels of HDAC6 in cells can suppress the contractile force of cell.

### 3.3 Transformed and invasive cells have higher traction force on the environment and reduced intracellular force on the nucleus compared to control cells, in a tubacin-dependent manner

**FIGURE 3:**
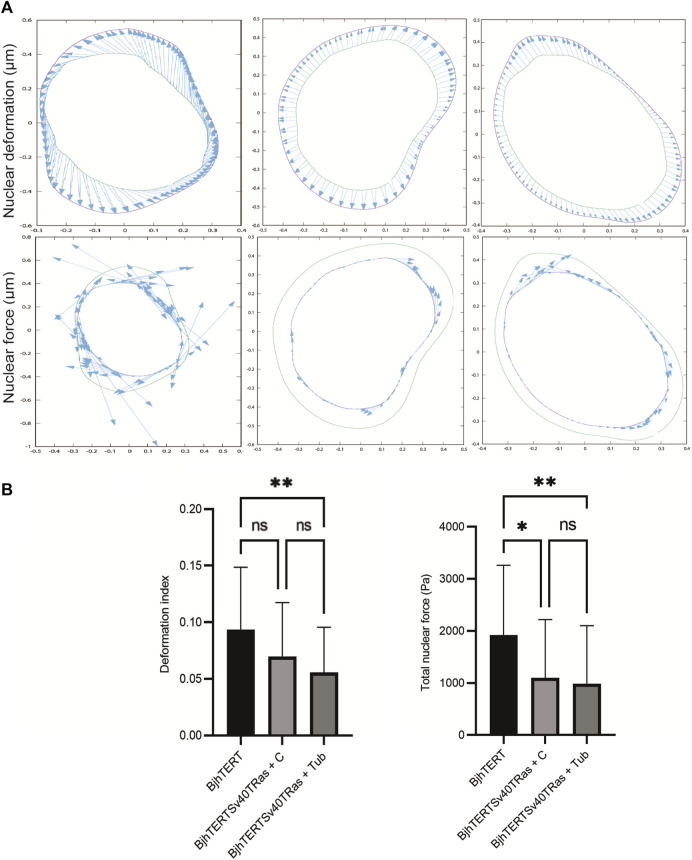
Transformed and invasive cells show lower intracellular forces on the nucleus. **(A)** Representative images of the nuclei of normal and transformed and invasive cells treated without or with tubacin (Tub), showing the nuclear deformation (top panel), with the deformed shape (magenta line), undeformed shape (green line) and deformation (cyan arrows), with each arrow scaled such that one unit of length on the axes represents a traction force of 250 Pa. and (bottom panel) the nuclear force with the undeformed shape (magenta line), deformed shape (green line) and traction forces (cyan arrows), with each arrow scaled such that one unit of length on the axes represents a traction force of 250 Pa .**(B)** Graphs of the deformation index (left) and the total nuclear force (right). Data from at least three independent biological repeats and presented as mean ± SD. *p ≤ 0.05, **p ≤ 0.01 (One way ANOVA tests). BjhTERT *n* = 26, BjhTERTSv40TRas + C *n* = 43, BjhTERTSv40TRas + Tub *n* = 41. Scale bar: 10 µm.

We next aimed to determine if the intracellular forces that cells exert on their nucleus is affected in transformed and invasive cells. We have previously observed that these transformed and invasive fibroblasts show a cytoskeleton that is less polymerised, coherent, extended and developed than in healthy cells (Rathje et al., 2014; Evans et al., 2022), and therefore hypothesised that transformed and invasive cells would exert reduced contractile forces on their nuclei. Indeed, we observed that the deformation of the cell nucleus, and the forces that the nucleus was subjected to were reduced in transformed and invasive cells, as compared to control, with no additional change by tubacin (Figure 3), and no effect of siRNA-mediated HDAC6 knockdown (Supplementary Figure S2). We did not observe any differences of the elastic stiffness of the nuclear material, when measured on the control cells without or with oncogene-expression using collodial probe force-mode atomic force microscopy (Supplementary Figure S3). It is therefore of particular note that the decrease in the deforming force was observed even without presuming that the nuclear material became softer. Because we have previously observed that oncogene-expression results in a more collapsed and perinuclear localisation of vimentin filaments and thinner and smaller F-actin stress fibres and cell matrix adhesions (Rönnlund et al., 2013; Rathje et al., 2014), we hypothesised that the coordination and correlation of intracellular and extracellular contractile forces of cells was altered in transformed and invasive cells. When analysing individual cells in the cell populations, we observed in all conditions that the forces on the environment and the nucleus were weakly positively correlated, with the exception of HDAC6 siRNA-treated cells (Supplementary Figure S4).

### 3.4 Loss of the HDAC6 protein reverses the reduced size and more circular shape of transformed and invasive cells to normal with no effect on cells migration speed and directionality

**FIGURE 4:**
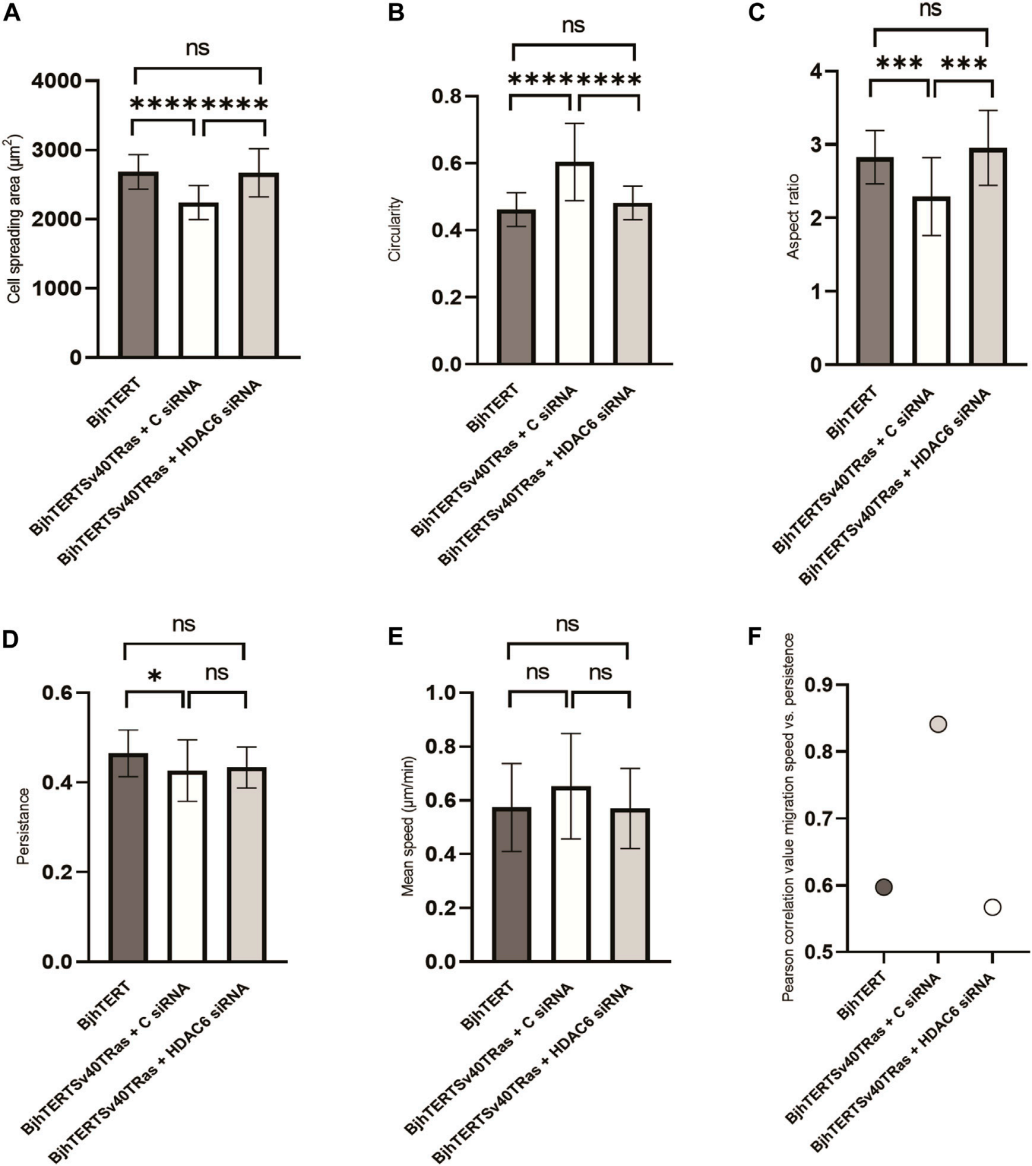
siRNA-mediated HDAC6 knockdown reverses the reduced spreading area, and the loss of the elongated cell shape of transformed and invasive cells back to normal, with no effect on cell migration, speed or persistence. The **(A)** cell spreading area, **(B)** cell circularity, **(C)** aspect ratio, **(D)** cell migration persistance, **(E)** cell migration mean speed, and **(F)** Pearson’s correlation between mean speed and persistence of fibroblasts treated with siRNA or controls, as indicated. Data from three independent biological repeats. *, *p* ≤ 0.05; **, *p* ≤ 0.01; ***, *p* ≤ 0.001; ****, *p* ≤ 0.0001 (One way ANOVA tests). BjhTERT *n* = 81, BjhTERTSv40TRas + C siRNA *n* = 81, BjhTERTSv40TRas + HDAC6 siRNA *n* = 81.

We then wished to determine if oncogene-induced changes in the morphology of cells require the HDAC6 protein. To this end, we analysed the cell spreading area, the cell shape, the persistence and mean speed of the migration of transformed and invasive cells on glass, upon siRNA-mediated knockdown of HDAC6. We observed that the transformed and invasive cells showed a reduced spreading area which was accompanied by a more circular and less elongated cell shape, as compared to normal cells (Figure 4). Knockdown of HDAC6 in the transformed and invasive cells reversed these phenotypes to that of the normal cells. The persistence of cell migration was decreased in transformed and invasive cells, with no change in the speed of cell migration. We further observed a correlation between cell speed and persistence in these cells (Figure 4). Although HDAC6 knockdown did not result in significant change in speed and persistence of cell migration, it reversed the correlation between speed and persistence of the transformed and invasive cells to normal (Figure 4). Taken together, these observations indicate that the capacity of fibroblasts to metastasise is linked to a reduced capacity to spread and form an elongated cells shape, and to decouple the speed from the directionality of cell migration, and that HDAC6 protein levels can be important for this control.

### 3.5 Transformed and invasive cells show reduced size and increased numbers of focal adhesions, phenotypes further enhanced upon inhibition of HDAC6 deacetylase activity or HDAC6 knockdown

**FIGURE 5:**
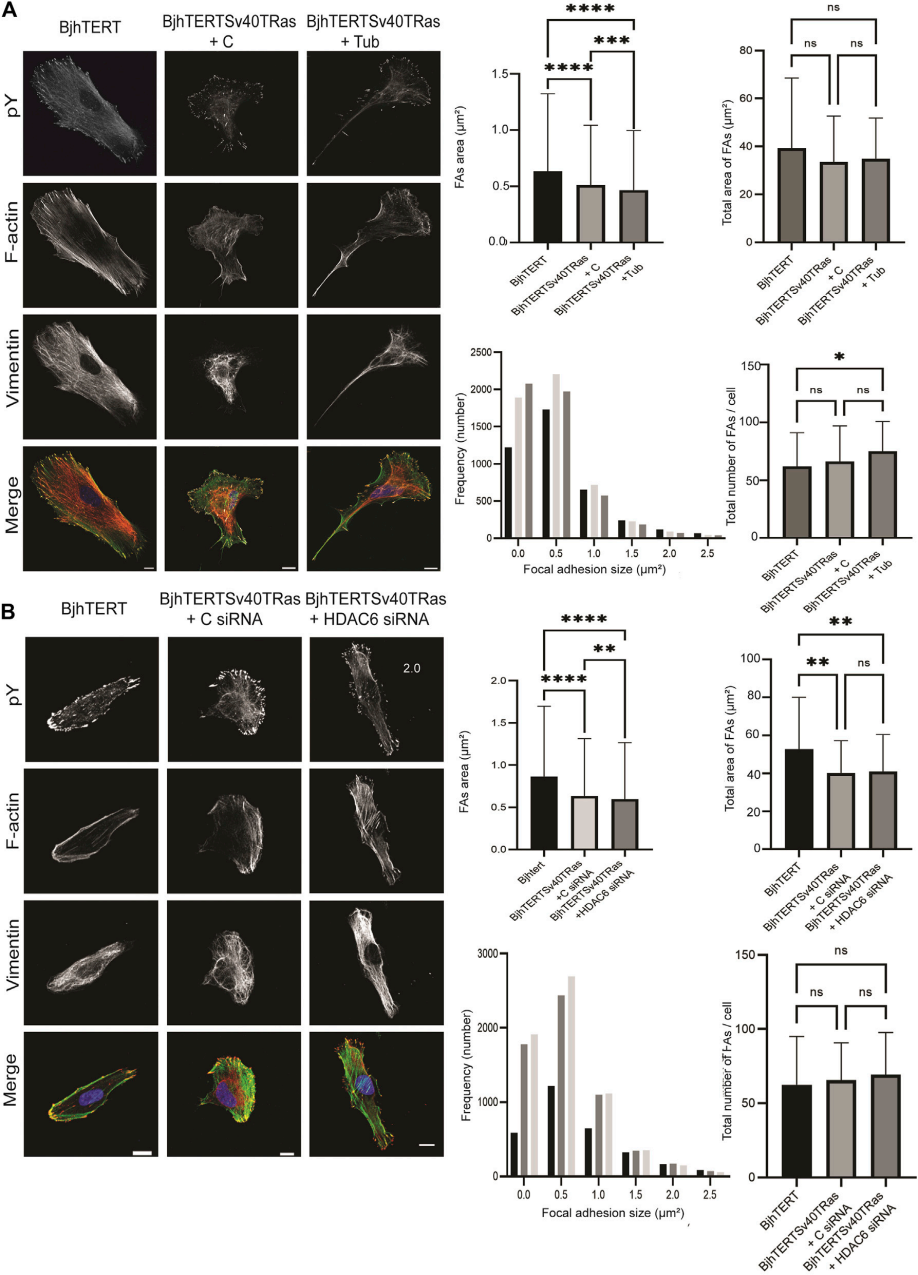
Transformed and invasive cells show smaller and increased number of focal adhesions, with further reduced sizes and increased numbers upon HDAC6 inhibition or HDAC6 knockdown. Left, representative images of normal, control or transformed and invasive cells treated with **(A)** tubacin (Tub) or DMSO control, or **(B)** HDAC6-targeting siRNA or control, showing phosphotyrosine (pY), F-actin, Vimentin, and merged images, as indicated, with quantification (right panel) of the average size of focal adhesions area and total size of focal adhesions area in μm^2^ (top panel) and frequency distribution of number of focal adhesions and total number of focal adhesions/cell (lower panel). Data is obtained from three independent biological repeats. *, *p* ≤ 0.05; **, *p* ≤ 0.01; ***, *p* ≤ 0.001; ****, *p* ≤ 0.0001 (One way ANOVA tests) (Frequency distribution test). For Figure 5A, BjhTERT *n* = 66, BjhTERTSv40TRas + C *n* = 76, BjhTERTSv40TRas + Tub *n* = 65. For Figure 5B, BjhTERT *n* = 49, BjhTERTSv40TRas + C siRNA *n* = 91, BjhTERTSv40TRas + HDAC6 siRNA *n* = 92.

**FIGURE 6:**
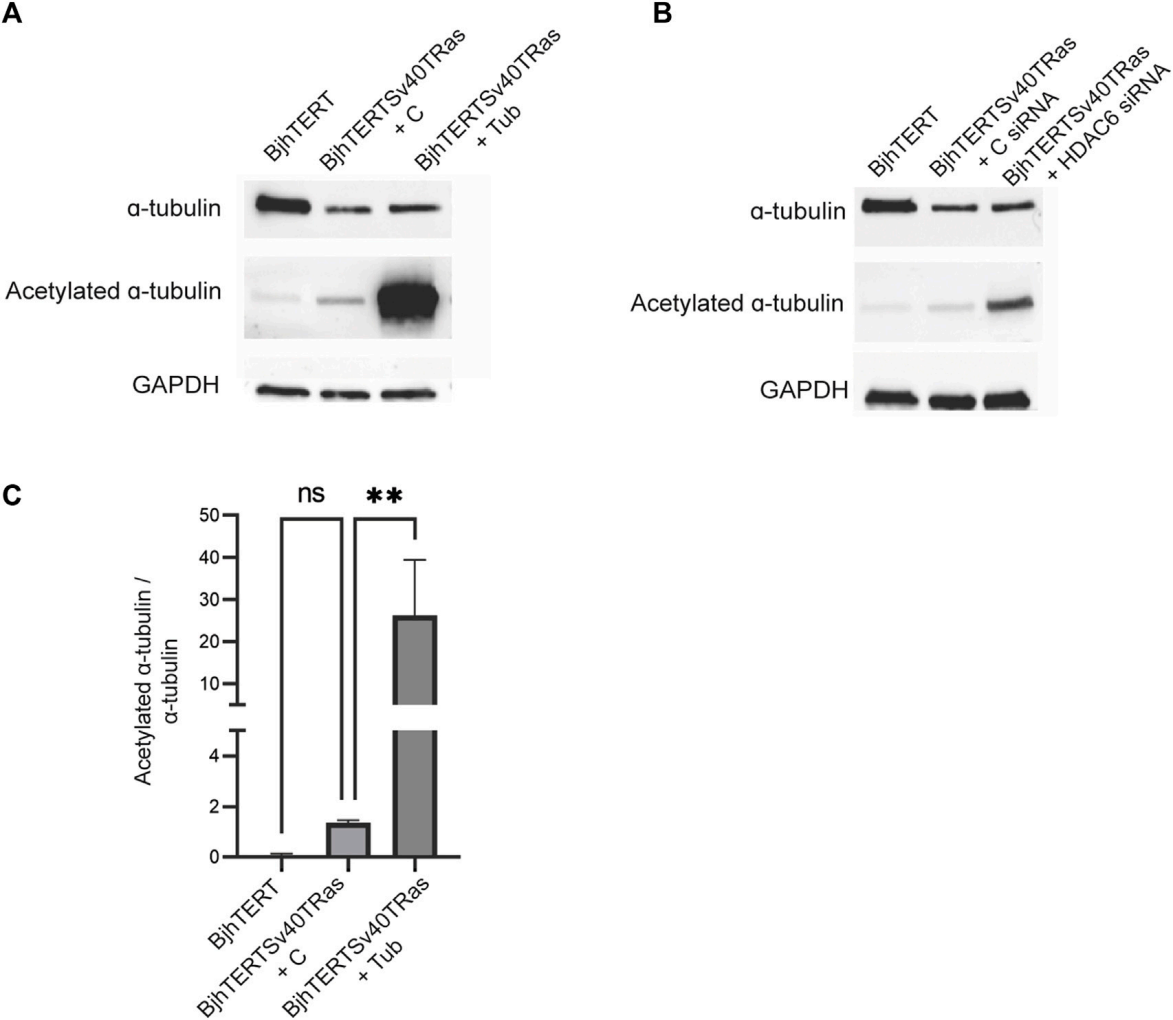
Tubacin and HDAC6 siRNA increase the acetylation of ɑ-tubulin in transformed and invasive fibroblasts. Normal and transformed and invasive cells treated without or with Tubacin **(A)**, or HDAC6 siRNA **(B)**, showing total acetylated tubulin and GAPDH loading control, as indicated with corresponding graph quantifying ratio between acetylated ɑ-tubulin and ɑ-tubulin treated without or with tubacin **(C)**. Data is obtained from three independent biological repeats **p ≤ 0.01.

Cellular traction forces are exerted through the formation of focal adhesions with the substrate (Bershadsky et al., 2006). To clarify the basis for HDAC6 effect on cancer contractile forces, we therefore compared the size and numbers of focal adhesions in the transformed and invasive cells cultured on glass, to normal control, and after tubacin-mediated inhibition of HDAC6 or HDAC6 knockdown in the transformed and invasive cells. Both HDAC6-inhibition and knockdown reduced the area of the focal adhesions (*p* ≤ 0.001, *p* ≤ 0.01 and *p* ≤ 0.0001 respectively), and showed an increased number of focal adhesions smaller than 1 um^2^ (Figure 5). Both the treatment with tubacin and with the HDAC6-targeting siRNA also increased the acetylation of tubulin (Figure 6). Taken together, this indicates that oncogenes, as well as the HDAC6-dependent tubulin acetylation suppress the growth and/or stability of mature focal adhesion.

## 4 Discussion

Our data shows that the contractile force that cells exert on their environment is increased in transformed and invasive cells, while the force that they exert on their nuclei is decreased, as compared to normal control cells. This highlights the possibility that these forces are less coordinated, and the cytoskeleton and the mechanical properties of the cell are less integrated in the oncogene-expressing, transformed and invasive cells than in normal control cells. This is in line with previous observations that the expression of oncogenes in the cells we use results in the formation of actin-based lamellipodia (Rathje et al., 2014; Evans et al., 2022), structures, which, in mechanical terms, are relatively separate from the cell body, and even able to detach and move away from the cell (Munevar et al., 2001b; Aspenström et al., 2004). Oncogene-expression also results in a less polymerised cytoskeleton, with more soluble, and less filamentous actin, tubulin and intermediate filaments (Rathje et al., 2014). The transformed and invasive cells further show a pronounced cytoskeletal reorganisation, which includes a significant increase of the curvature of vimentin filaments and a collapse of vimentin from the cell periphery to a vimentin cage around the nucleus (Rönnlund et al., 2013; Evans et al., 2022). Because the increased curvature of vimentin filaments can be used as a proxy for loss of physical force on the filaments (Smoler et al., 2020), this suggest that the cytoskeletal force-transduction in invasive cells is defective. The transformed and invasive cells have also lost the elongated, polarised cell shape, and the directionality of cell migration (Figures 4, 5,; Rathje et al., 2014; Evans et al., 2022). Taken together, this suggests that the force-transduction from cell-matrix adhesions to the nucleus is defective in the transformed and invasive cells. To our knowledge, our data provides the first direct comparison and correlation between the physical traction forces that individual cells exert on the surrounding environment to the forces they apply on their nucleus, and how these are defective in transformed and invasive cells.

Our findings are in line with previous reports that show that metastasising cancer cells exert stronger forces on their environment (Kraning-Rush et al., 2012). However, it should be noted that the traction forces cells have also been reported to not be a reliable marker for cell transformation and metastasis (Mierke et al., 2008). Epithelial and mesenchymal cell types have fundamentally different composition and spatial organisation of their cytoskeleton and cell-matrix adhesions. It is possible that the phenotypes we observed are specific to cancer cells of mesenchymal origin. Tumours like malignant mesothelioma, various sarcomas or dedifferentiated epithelial cancers that express vimentin and display mesenchymal phenotypes can therefore be used for a future validation of this phenomena in cancer cells. We further observed that the loss of cell attachment reduced the forces on the nucleus, which is consistent with the concept that the anchorage of the cytoskeleton to the extracellular matrix via cell-matrix adhesions is important for cells to generate intracellular cytoplasmic force on organelles. The transformed and invasive cells showed an increased level of HDAC6, and the specific inhibition of HDAC6 activity by tubacin in these cells reversed the increased maximum forces to normal. We further confirmed that in our system, tubacin did not change the protein levels of HDAC6 (Supplementary Figure S5). We have previously shown that the selective inhibition of HDAC6 activity by tubacin in these cells reverses the cell spreading, shape and cell migration to normal (Evans et al., 2022). In this present study, we observe the same or similar effects upon siRNA-mediated HDAC6 knockdown. These observations are in line with our earlier observations that expression of the oncogenes c-Myc and SV40T in normal fibroblasts induces the HDAC6 protein levels, an HDAC6-dependent reorganisation of actin and vimentin filaments, and the stiffness of cells (Rathje et al., 2014). The size of cell-matrix adhesions was reduced by oncogene-expression, and not reversed, but rather further reduced by the loss of HDAC6 function and protein levels. Although both tubacin and siRNA-mediated HDAC6 knockdown increased the acetylation of alpha-tubulin, the effects on cellular force were opposite. A similar discrepancy has been observed in microtubule dynamics, where tubacin inhibition, but not siRNA-mediated HDAC6 knockdown reduces the growth and shrinkage velocities of microtubules, although both treatments increase tubulin acetylation (Zilberman et al., 2009). This study showed that HDAC6 is associated with microtubule plus-end linked proteins, and suggests that the presence of a HDAC6 protein with impaired catalytic activity, and not HDAC6-mediated deacetylation of tubulin, controls microtubule dynamics. Upon inhibition of HDAC6 by tubacin, HDAC6 would fail to dissociate from microtubules, resulting in the capping of the growing end of microtubules, which is known to suppress microtubule polymerisation and dynamics (Zilberman et al., 2009; Asthana et al., 2013). These results are in line with the hypothesis that cellular contractile force requires the binding of a functional HDAC6 to the plus end of microtubules which allows microtubule dynamics, and that HDAC6-dependent microtubule deacetylation suppresses the growth of focal adhesions and consequently the spreading, the elongated shape of cells, and the cell migration speed and directionality. Because microtubule acetylation is linked to an increased capacity of kinesin to transport proteins in the anterograde direction, toward the periphery of cells, on microtubules (Reed et al., 2006), we suggest that HDAC6-induced loss of microtubule acetylation prevents the delivery of proteins required for the maturation and growth of focal adhesions (Supplementary Figure S6). We do not exclude the possibility that HDAC6 can control acto-myosin contractile force via other substrate than tubulin, such as HSP90 and cortactin (Martin and Leibovich, 2005; Zhang et al., 2007). Cortactin promotes F-actin polymerisation and branching, resulting in the lamellipodia at the cell leading edges, which been linked to cancer invasiveness (Kirkbride et al., 2011), and the generation of traction forces of cells (Gad et al., 2012). Hence, the opposite effect we observe by HDAC6 inhibition or knockdown on extracellular traction forces can therefore also be due to that while HDAC6-mediated deacetylation of these substrates control the acto-myosin contractile mechanisms directly, the microtubule-binding capacity of HDAC6 exert an indirect, microtubule-mediated effect on acto-myosin generated force.

Our observation that the transformed and invasive cells showed increased forces on their surrounding environment, without an increased focal adhesion size or number, highlight the possibility that the size of focal adhesions or number cannot be used as a proxy for the level of transmitted force. This is in line with our previous observations in fibroblasts that rather link increased forces to the dissolution of large focal adhesion and rather to the increased density of nanoscale adhesions in the nanometer range, and dense bundles of very thin actin-fibres (Gad et al., 2012). This highlights the possibility that focal adhesion turnover, increased assembly, and/or reduced disassembly can regulate forces of cells. It should also be noted that a stiff underlying surface induces more pronounced cell-matrix adhesions and cytoskeletal structures than soft, and most research in cell adhesion, cytoskeleton, HDAC6 and migration is based on cells cultured on plastic or glass. To allow the maximal sensitivity of the analysis, and comparison to the previous knowledge in the field, we therefore performed all analyses on glass or plastic, except for the traction force measurements which, as required by the method, were performed on gels. Future analysis of cells on surfaces of different stiffness and structures would be of interest to clarify the role of oncogenes and HDAC6 in mechanosensation and -response. Our data highlight the possibility that the regulation of oncogenes on cell forces and cell migration, and the cytoskeleton and cell-matrix adhesions is governed by a balance between separate signalling pathways, including the separate deacetylase and protein-binding functions of HDAC6.

## 5 Conclusion

Our findings suggest that the cell transformation and invasion is accompanied by an increased physical force that cells exert on their environment, combined with a decreased intracellular cytoplasmic force on the cell nucleus, by a mechanism that requires HDAC6. The data is consistent with the hypothesis that oncogenes, via the separate deacetylase-, and protein-binding functions of HDAC6, regulate the physical forces that cells exert on their environment and nucleus, their cell-matrix adhesions, the cell spreading, shape, migration which promote the motility and invasion of cells in cancer.

## Data Availability

The original contributions presented in the study are included in the article/Supplementary Material, further inquiries can be directed to the corresponding author.

## References

[B1] Alisafaei F. Jokhun D. S. Shivashankar G. V. Shenoy V. B. (2019). Regulation of nuclear architecture, mechanics, and nucleocytoplasmic shuttling of epigenetic factors by cell geometric constraints. Proc. Natl. Acad. Sci. U. S. A. 116, 13200–13209. 10.1073/pnas.1902035116 31209017 PMC6613080

[B2] Aspenström P. Fransson Å. Saras J. (2004). Rho GTPases have diverse effects on the organization of the actin filament system. Biochem. J. 377 (Pt 2), 327–337. 10.1042/BJ20031041 14521508 PMC1223866

[B3] Asthana J. Kapoor S. Mohan R. Panda D. (2013). Inhibition of HDAC6 deacetylase activity increases its binding with microtubules and suppresses microtubule dynamic instability in MCF-7 cells. J. Biol. Chem. 288, 22516–22526. 10.1074/jbc.M113.489328 23798680 PMC3829339

[B4] Bance B. Seetharaman S. Leduc C. Boeda B. Etienne-Manneville S. (2019). Microtubule acetylation but not detyrosination promotes focal adhesion dynamics and astrocyte migration. J. Cell Sci. 132, jcs225805. 10.1242/jcs.225805 30858195

[B5] Bershadsky A. D. Ballestrem C. Carramusa L. Zilberman Y. Gilquin B. Khochbin S. (2006). Assembly and mechanosensory function of focal adhesions: Experiments and models. Eur. J. Cell Biol. 85, 165–173. 10.1016/j.ejcb.2005.11.001 16360240

[B6] Binker M. G. Zhao D. Y. Pang S. J. Harrison R. E. (2007). Cytoplasmic linker protein-170 enhances spreading and phagocytosis in activated macrophages by stabilizing microtubules. J. Immunol. 179, 3780–3791. 10.4049/jimmunol.179.6.3780 17785815

[B7] Burridge M. C.-W. A. K. Burridge K. (1996). Rho-stimulated contractility drives the formation of stress fibers and focal adhesions. J. Cell Biol. 133, 1403–1415. 10.1083/jcb.133.6.1403 8682874 PMC2120895

[B8] Caille N. Thoumine O. Tardy Y. Meister J. (2002). Contribution of the nucleus to the mechanical properties of endothelial cells. *J. Biomechanics*, Feb 35 (2), 177–187. 10.1016/s0021-9290(01)00201-9 11784536

[B9] Carl P. Schillers H. (2008). Elasticity measurement of living cells with an atomic force microscope: Data acquisition and processing. Pflugers Arch. 457, 551–559. 10.1007/s00424-008-0524-3 18481081

[B10] Chiotaki R. Polioudaki H. Theodoropoulos P. A. (2014). Differential nuclear shape dynamics of invasive andnon-invasive breast cancer cells are associated with actin cytoskeleton organization and stability. Biochem. Cell Biol. 92, 287–295. 10.1139/bcb-2013-0120 25053513

[B11] Clevers J. E. V. A. H. Clevers H. (2016). Tissue-specific designs of stem cell hierarchies. Nat. Cell Biol. 18, 349–355. 10.1038/ncb3332 26999737

[B12] Danielsson F. Skogs M. Huss M. Rexhepaj E. O'Hurley G. Klevebring D. (2013). Majority of differentially expressed genes are down-regulated during malignant transformation in a four-stage model. Proc. Natl. Acad. Sci. U. S. A. 110, 6853–6858. 10.1073/pnas.1216436110 23569271 PMC3637701

[B13] Deb Roy A. Gross E. G. Pillai G. S. Seetharaman S. Etienne-Manneville S. Inoue T. (2022). Non-catalytic allostery in α-TAT1 by a phospho-switch drives dynamic microtubule acetylation. J. Cell Biol. 221, e202202100. 10.1083/jcb.202202100 36222836 PMC9565784

[B14] Dos Santos A. Cook A. W. Gough R. E. Schilling M. Olszok N. A. Brown I. (2021). DNA damage alters nuclear mechanics through chromatin reorganization. Nucleic Acids Res. 49, 340–353. 10.1093/nar/gkaa1202 33330932 PMC7797048

[B15] Dos Santos A. Toseland C. P. (2021). Regulation of nuclear mechanics and the impact on DNA damage. Int. J. Mol. Sci. 22, 3178. 10.3390/ijms22063178 33804722 PMC8003950

[B16] Estabrook I. D. Thiam H. R. Piel M. Hawkins R. J. (2021). Calculation of the force field required for nucleus deformation during cell migration through constrictions. PLoS Comput. Biol. 17, e1008592. 10.1371/journal.pcbi.1008592 34029312 PMC8177636

[B17] Evans C. A. Kim H. R. Macfarlane S. C. Nowicki P. I. A. Baltes C. Xu L. (2022). Metastasising fibroblasts show an HDAC6-dependent increase in migration speed and loss of directionality linked to major changes in the vimentin interactome. Int. J. Mol. Sci. 23, 1961. 10.3390/ijms23041961 35216078 PMC8880509

[B18] Feld L. Kellerman L. Mukherjee A. Livne A. Bouchbinder E. Wolfenson H. (2020). Cellular contractile forces are nonmechanosensitive. Sci. Adv. 6 (17), eaaz6997–9. 10.1126/sciadv.aaz6997 32494649 PMC7176410

[B19] Franco-Barazza J. Beacham D. A. Amatangelo M. D. Cukierman E. (2016). Preparation of extracellular matrices produced by cultured and primary fibroblasts. Curr. Protoc. Cell Biol. 71 (1), 10.9.1–10.9.34. 10.1002/cpcb.2 PMC5058441 27245425

[B20] Gad A. K. Ronnlund D. Spaar A. Savchenko A. A. Petranyi G. Blom H. (2012). Rho GTPases link cellular contractile force to the density and distribution of nanoscale adhesions. FASEB J. 26, 2374–2382. 10.1096/fj.11-195800 22371528

[B21] Ghassemi S. Meacci G. Liu S. Gondarenko A. A. Mathur A. Roca-Cusachs P. (2012). Cells test substrate rigidity by local contractions on submicrometer pillars. Proc. Natl. Acad. Sci. U. S. A. 109, 5328–5333. 10.1073/pnas.1119886109 22431603 PMC3325713

[B22] Guilak F. Tedrow J. R. Burgkart R. (2000). Viscoelastic properties of the cell nucleus. Biochem. Biophys. Res. Commun. 269, 781–786. 10.1006/bbrc.2000.2360 10720492

[B23] Haggarty J, S. Koeller K. M. Wong J, C. Grozinger C, M. Schreiber S, L. (2003b). Domain-selective small-molecule inhibitor of histone deacetylase 6 (HDAC6)-mediated tubulin deacetylation. PNAS 100, 4389–4394. 10.1073/pnas.0430973100 12677000 PMC153564

[B24] Haggarty S. J. Koeller K. M. Wong J. C. Butcher R. A. Schreiber S. L. (2003a). Multidimensional chemical genetic analysis of diversity-oriented synthesis-derived deacetylase inhibitors using cell-based assays. Chem. Biol. 10, 383–396. 10.1016/s1074-5521(03)00095-4 12770821

[B25] Hahn W. C. Counter C. M. Lundberg A. S. Beijersbergen R. L. Brooks M. W. Weinberg R. A. (1999). Creation of human tumour cells with defined genetic elements. NATURE 400, 464–468. 10.1038/22780 10440377

[B26] Holenstein C. N. Horvath A. Schar B. Schoenenberger A. D. Bollhalder M. Goedecke N. (2019). The relationship between metastatic potential and *in vitro* mechanical properties of osteosarcoma cells. Mol. Biol. Cell 30, 887–898. 10.1091/mbc.E18-08-0545 30785850 PMC6589788

[B27] Horwitz A. R. (2012). The origins of the molecular era of adhesion research. Nat. Rev. Mol. Cell Biol. 13, 805–811. 10.1038/nrm3473 23151664 PMC3692278

[B28] Hu Z. Wei F. Su Y. Wang Y. Shen Y. Fang Y. (2023). Histone deacetylase inhibitors promote breast cancer metastasis by elevating NEDD9 expression. Signal Transduct. Target Ther. 8, 11. 10.1038/s41392-022-01221-6 36604412 PMC9816171

[B29] Jain N. Iyer K. V. Kumar A. Shivashankar G. V. (2013). Cell geometric constraints induce modular gene-expression patterns via redistribution of HDAC3 regulated by actomyosin contractility. Proc. Natl. Acad. Sci. U. S. A. 110, 11349–11354. 10.1073/pnas.1300801110 23798429 PMC3710882

[B30] Jiu Y. (2018). Vimentin intermediate filaments function as a physical barrier during intracellular trafficking of caveolin-1. Biochem. Biophys. Res. Commun. 507, 161–167. 10.1016/j.bbrc.2018.10.199 30415776

[B31] Jiu Y. Lehtimaki J. Tojkander S. Cheng F. Jaalinoja H. Liu X. (2015). Bidirectional interplay between vimentin intermediate filaments and contractile actin stress fibers. Cell Rep. 11, 1511–1518. 10.1016/j.celrep.2015.05.008 26027931

[B32] Jiu Y. Peranen J. Schaible N. Cheng F. Eriksson J. E. Krishnan R. (2017). Vimentin intermediate filaments control actin stress fiber assembly through GEF-H1 and RhoA. J. Cell Sci. 130, 892–902. 10.1242/jcs.196881 28096473 PMC5358333

[B33] Karen A. Beningo M. D. Irina K. Victor Small J. Wang Y. U-L. I. (2001). Nascent focal adhesions are responsible for the generation. JCB 153, 881–887. 10.1083/jcb.153.4.881 11352946 PMC2192381

[B34] Katiyar A. Zhang J. Antani J. D. Yu Y. Scott K. L. Lele P. P. 2022. The nucleus bypasses obstacles by deforming like a drop with surface tension mediated by lamin A/C. *Adv. Sci. (Weinh),* Aug 9(23), 1–14. 10.1002/advs.202201248 PMC9376816 35712768

[B35] Kim H. R. Warrington S. J. Lopez-Guajardo A. Al Hennawi K. Cook S. L. Griffith Z. D. J. (2022). ALD-R491 regulates vimentin filament stability and solubility, cell contractile force, cell migration speed and directionality. Front. Cell Dev. Biol. 10, 926283. 10.3389/fcell.2022.926283 36483676 PMC9723350

[B36] Kirkbride K. C. Sung B. H. Sinha S. Weaver A. M. (2011). Cortactin: A multifunctional regulator of cellular invasiveness. Cell Adh Migr. 5 (2), 187–198. 10.4161/cam.5.2.14773 21258212 PMC3084985

[B37] Kraning-Rush C. M. Califano J. P. Reinhart-King C. A. 2012. Cellular traction stresses increase with increasing metastatic potential. PLoS One, 7(2), 325722-e32610. 10.1371/journal.pone.0032572 PMC3289668 22389710

[B38] Krendel M. Zenke F. T. Bokoch G. M. (2002). Nucleotide exchange factor GEF-H1 mediates cross-talk between microtubules and the actin cytoskeleton. Nat. Cell Biol. 4 (4), 294–301. 10.1038/ncb773 11912491

[B39] Lafarga V. Aymerich I. Tapia O. Mayor F. JR. Penela P. (2012). A novel GRK2/HDAC6 interaction modulates cell spreading and motility. EMBO J. 31, 856–869. 10.1038/emboj.2011.466 22193721 PMC3280560

[B40] Lammerding J. (2011). Mechanics of the nucleus. Compr. Physiol. 1, 783–807. 10.1002/cphy.c100038 23737203 PMC4600468

[B41] Lee S. Kumar S. (2020). Cofilin is required for polarization of tension in stress fiber networks during migration. J. Cell Sci. 133, jcs243873. 10.1242/jcs.243873 32501289 PMC7358140

[B42] Lee Y. S. Lim K. H. Guo X. Kawaguchi Y. Gao Y. Barrientos T. (2008). The cytoplasmic deacetylase HDAC6 is required for efficient oncogenic tumorigenesis. Cancer Res. 68, 7561–7569. 10.1158/0008-5472.CAN-08-0188 18794144 PMC2978070

[B43] Li D. Sun X. Zhang L. Yan B. Xie S. Liu R. (2014). Histone deacetylase 6 and cytoplasmic linker protein 170 function together to regulate the motility of pancreatic cancer cells. Protein Cell 5 (3), 214–223. 10.1007/s13238-013-0010-3 24474193 PMC3967059

[B44] Ling L. Hu F. Ying X. Ge J. Wang Q. (2018). HDAC6 inhibition disrupts maturational progression and meiotic apparatus assembly in mouse oocytes. Cell Cycle 17, 550–556. 10.1080/15384101.2017.1329067 28598228 PMC5969567

[B45] Martiel J. L. Leal A. Kurzawa L. Balland M. Wang I. Vignaud T. (2015). Measurement of cell traction forces with ImageJ. Methods Cell Biol. 125, 269–287. 10.1016/bs.mcb.2014.10.008 25640434

[B46] Martin P. Leibovich S. J. (2005). Inflammatory cells during wound repair: The good, the bad and the ugly. Trends Cell Biol. 15 (11), 599–607. 10.1016/j.tcb.2005.09.002 16202600

[B47] Mierke C. T. Rosel D. Fabry B. Brabek J. (2008). Contractile forces in tumor cell migration. Eur. J. Cell Biol. 87 (8-9), 669–676. 10.1016/j.ejcb.2008.01.002 18295931 PMC2566782

[B48] Munevar S. Wang Y.-L, W. Dembo M. (2001a). Traction force microscopy of migrating normal and H-ras transformed 3T3 fibroblasts. Biophysical J. 80, 1744–1757. 10.1016/s0006-3495(01)76145-0 PMC1301364 11259288

[B49] Munevar S. Wang Y. Dembo M. (2001b). Distinct roles of frontal and rear cell-substrate adhesions in fibroblast migration. Mol. Biol. Cell 12 (12), 3947–3954. 10.1091/mbc.12.12.3947 11739792 PMC60767

[B50] Parsons J. T. Horwitz A. R. Schwartz M. A. (2010). Cell adhesion: Integrating cytoskeletal dynamics and cellular tension. Nat. Rev. Mol. Cell Biol. 11, 633–643. 10.1038/nrm2957 20729930 PMC2992881

[B51] Rafiq N. B. M. Nishimura Y. Plotnikov S. V. Thiagarajan V. Zhang Z. Shi S. (2019). A mechano-signalling network linking microtubules, myosin IIA filaments and integrin-based adhesions. Nat. Mater 18, 638–649. 10.1038/s41563-019-0371-y 31114072

[B52] Rathje L. S. Nordgren N. Pettersson T. Rönnlund D. Widengren J. Aspenstrom P. (2014). Oncogenes induce a vimentin filament collapse mediated by HDAC6 that is linked to cell stiffness. Proc. Natl. Acad. Sci. U. S. A. 111, 1515–1520. 10.1073/pnas.1300238111 24474778 PMC3910606

[B53] Reed N. N. Cai D. Blasius T. L. Jih G. T. Meyhofer E. Gaertig J. 2006, Microtubule acetylation promotes kinesin-1 binding and transport. Curr. Biol. Nov. 7 (21):2166–2172. 10.1016/j.cub.2006.09.014 17084703

[B54] Ren Y. Li R. Zheng Y. Busch H. (1998). Cloning and characterization of GEF-H1, a microtubule-associated guanine nucleotide exchange factor for Rac and Rho GTPases. J. Biol. Chem. 273, 34954–34960. 10.1074/jbc.273.52.34954 9857026

[B55] Rönnlund D. Gad A. K. Blom H. Aspenstrom P. Widengren J. (2013). Spatial organization of proteins in metastasizing cells. Cytom. A 83, 855–865. 10.1002/cyto.a.22304 23657948

[B56] Schape J. Prausse S. Radmacher M. Stick R. (2009). Influence of lamin A on the mechanical properties of amphibian oocyte nuclei measured by atomic force microscopy. Biophys. J. 96, 4319–4325. 10.1016/j.bpj.2009.02.048 19450502 PMC2712154

[B57] Schreiner S. M. Koo P. K. Zhao Y. Mochrie S. G. King M. C. (2015). The tethering of chromatin to the nuclear envelope supports nuclear mechanics. Nat. Commun. 6, 7159. 10.1038/ncomms8159 26074052 PMC4490570

[B58] Seetharaman S. Vianay B. Roca V. Farrugia A. J. De Pascalis C. Boeda B. (2022). Microtubules tune mechanosensitive cell responses. Nat. Mater 21, 366–377. 10.1038/s41563-021-01108-x 34663953

[B59] Smoler M. Coceano G. Testa I. Bruno L. Levi V. (2020). Apparent stiffness of vimentin intermediate filaments in living cells and its relation with other cytoskeletal polymers. Biochimica Biophysica Acta (BBA) 1867 (8), 118726. 10.1016/j.bbamcr.2020.118726 32320724

[B60] Stivarou T. Stellas D. Vartzi G. Thomaidou D. Patsavoudi E. (2016). Targeting highly expressed extracellular HSP90 in breast cancer stem cells inhibits tumor growth *in vitro* and *in vivo* . Cancer Biol. Ther. 17, 799–812. 10.1080/15384047.2016.1195041 27259689 PMC5004692

[B61] Sun B. Meizhen C. Hawks C. L. Pereira-Smith O. M. Hornsby P. J. (2005). The minimal set of genetic alterations required for conversion of primary human fibroblasts to cancer cells in the subrenal capsule assay. Neoplasia 7 (6), 585–593. 10.1593/neo.05172 16036109 PMC1501282

[B62] Swift J. Ivanovska I. L. Buxboim A. Harada T. Dingal P. C. Pinter J. (2013). Nuclear lamin-A scales with tissue stiffness and enhances matrix-directed differentiation. Science 341, 1240104. 10.1126/science.1240104 23990565 PMC3976548

[B63] Teo J. L. Lim C. T. Yap A. S. Saw T. B. (2020). A biologist’s guide to traction force microscopy using polydimethylsiloxane substrate for two-dimensional cell cultures. Star. Protoc. 1 (2), 100098. 10.1016/j.xpro.2020.100098 33111126 PMC7580222

[B64] Terriac E. Schultz S. Lautenschläger F. (2019). Vimentin intermediate filament rings deform the nucleus during the first steps of adhesion. Front. Cell Dev. Biol. 7, 106. 10.3389/fcell.2019.00106 31263698 PMC6590062

[B65] Trichet L. Le Digabel J. Hawkins R. J. Vedula S. R. Gupta M. Ribrault C. (2012). Evidence of a large-scale mechanosensing mechanism for cellular adaptation to substrate stiffness. Proc. Natl. Acad. Sci. U. S. A. 109, 6933–6938. 10.1073/pnas.1117810109 22509005 PMC3344951

[B66] Zhang X. Yuan Z. Zhang Y. Yong S. Salas-Burgos A. Koomen J. (2007). HDAC6 modulates cell motility by altering the acetylation level of cortactin. Mol. Cell 27, 197–213. 10.1016/j.molcel.2007.05.033 17643370 PMC2684874

[B67] Zilberman Y. Ballestrem C. Carramusa L. Mazitschek R. Khochbin S. Bershadsky A. (2009). Regulation of microtubule dynamics by inhibition of the tubulin deacetylase HDAC6. J. Cell Sci. 122, 3531–3541. 10.1242/jcs.046813 19737819

