# Supplementary material for: Regulation of cellular contractile force, shape and migration of fibroblasts by oncogenes and Histone deacetylase 6
Source: Front Mol Biosci. 2023 Jul 20;10:1197814. doi: 10.3389/fmolb.2023.1197814 (PMC10411354; doi:10.3389/fmolb.2023.1197814)
Supplement: Supplementary file 1 [file DataSheet1.docx]

***Supplementary materials***

Regulation of Cellular Contractile Force, Shape and Migration of Fibroblasts by Oncogenes and Histone deacetylase 6

**Ana López-Guajardo, Azeer Zafar, Khairat Al Hennawi, Valentina Rossi, Abdulaziz Alrwaili, Jessica D. Medcalf, Mark Dunning, Niklas Nordgren, Torbjörn Pettersson, Ian D. Estabrook, Rhoda J. Hawkins, Annica K. B. Gad***

*** Correspondence:** Corresponding Author: [annica.gad.2@ki.se](mailto:annica.gad.2@ki.se)


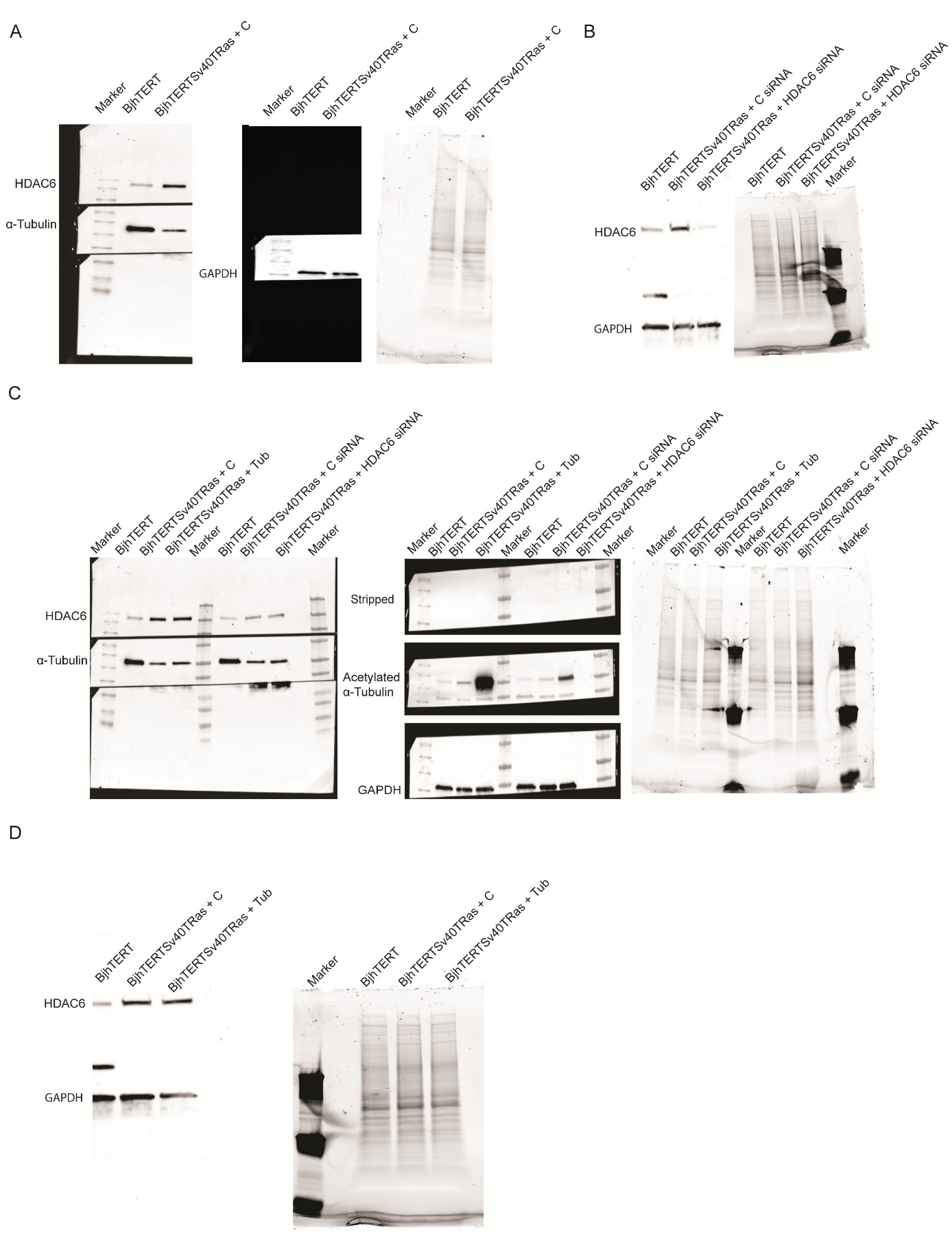


**Supplementary Figure 1. Representatives Western blot membranes**. Western blot membrane showing the source membranes for the blots shown in the manuscript figures (as indicated within brackets) and the total proteins on the membrane. **(A)** membrane showing levels of analysed proteins (left) and loading control (middle) and total proteins (right) (for Fig. 1A). **(B)** membrane of the proteins (left) and the total proteins on the membrane (right) (for Fig. 2A). **(C)** membrane showing the analysed proteins (left), the stripped alpha-tubulin membrane (middle, top image), the same membrane re-probed against acetylated tubulin (middle, middle panel), and, after a second stripping, against GAPDH loading control (bottom, middle panel), with the total proteins (right) (for Fig. 6A and 6B). **(D)** Blot of indicated proteins (left) and the total proteins (right) (for Suppl. Fig. 5).

**Supplementary Text 1. Script in R to sum the modulus for the nuclear forces.** The total force that cells exerted on their nuclei was calculated from output files by summing the modulus of the force vectors over the perimeter of the cell using the R script described below:

library(tidyverse)

files <- list.files(".", pattern = "force.txt",full.names = TRUE, recursive = TRUE)

meta <- data.frame(stringr::str_split_fixed(files, "/",n = 6))

meta$samplename <- paste(meta[,4], meta[,5],sep="_")

meta$files <- files

forces <- do.call("rbind", lapply(files, function(x) read_delim(x, delim = " ", skip = 1, col_names = FALSE) %>% mutate(file = x) %>% rename(x = X1, y = X2, Tx = X3, Ty = X4)))

forces$Sample <- meta$samplename[match(forces$file, meta$files)]

forces <- mutate(forces, Magnitude = sqrt(Tx^2 + Ty^2), x_diff = x - lag(x), y_diff = y - lag(y), Dist = sqrt(x_diff^2 + y_diff^2), Force = Dist * Magnitude) %>%

select(-file)

*Note: columns in input file force.txt are as follows: x pos y pos Tx Ty*

**Supplementary Text 2. Script in R to calculate a deformation index for the calculated deformations.** The deformation of each nucleus was calculated from output files by summing the modulus of the deformation vectors and dividing by the perimeter of the undeformed nucleus using the R script described below:

library(tidyverse)

files <- list.files(".", pattern = "deformation.txt",full.names = TRUE, recursive = TRUE)

index <- 1

perimeter <- rep(0, length(files))

file_numlines <- rep(0, length(files))

perimeter_source <- rep(0, length(files))

deformation_sum_magnitude <- rep(0, length(files))

index_source_new <- rep(0, length(files))

deformation_mean <- rep(0, length(files))

for (i in files) {

temp_data <- read_delim(i, delim = " ", skip = 1, col_names = FALSE, show_col_types = FALSE)

if (index>1)

{ file_numlines[index]=file_numlines[index-1]+lengths(temp_data)[[1]] }

else{ file_numlines[index]=lengths(temp_data)[[1]] }

source_x=temp_data$X1

source_y=temp_data$X2

target_x=temp_data$X4

target_y=temp_data$X5

diff_x_source=diff(source_x)

diff_y_source=diff(source_y)

perimeter_source[index]=sum(sqrt(diff_x_source^2+diff_y_source^2))+sqrt((source_x[1]-source_x[length(source_x)])^2+(source_y[1]-source_y[length(source_y)])^2)

deformation_sum_magnitude[index]=sum(sqrt(temp_data$X7^2+temp_data$X8^2))

deformation_mean[index]=deformation_sum_magnitude[index]/lengths(temp_data)[[1]];

index_source_new[index] = deformation_mean[index] / perimeter_source[index]

index <- index +1

}

meta <- data.frame(stringr::str_split_fixed(files, "/",n = 6))

meta$samplename <- paste(meta[,4], meta[,5],sep="_")

meta$files <- files

deformation <- do.call("rbind", lapply(files, function(x) read_delim(x, delim = " ", skip = 1, col_names = FALSE,show_col_types = FALSE)

%>% mutate(file = x) #sets file name to x...

%>% rename(source_x = X1, # Renaming parameters.

source_y = X2,

source_z = X3,

target_x = X4,

target_y = X5,

target_z = X6,

dx = X7,

dy = X8,

dz = X9)

)

)

deformation$Sample <- meta$samplename[match(deformation$file, meta$files)]

deformation <- mutate(deformation, Magnitude = sqrt(dx^2 + dy^2))

deformation <- mutate(deformation, deformation_sum_magnitude = 0)

deformation <- mutate(deformation, deformation_mean=0)

deformation <- mutate(deformation, Perimeter_source = 0)

deformation <- mutate(deformation, index_source_new =0)

for (i in 1:(length(file_numlines)))

{ if (i==1)

{ start_index = 1

end_index=file_numlines[1]

}

else

{ start_index=file_numlines[i-1]+1

end_index=file_numlines[i]

}

for (j in start_index:end_index )

{

deformation$Perimeter_source[j]=perimeter_source[i]

deformation$deformation_sum_magnitude[j]=deformation_sum_magnitude[i]

deformation$deformation_mean[j]=deformation_mean[i]

deformation$index_source_new[j] =index_source_new[i]

}

}

deformation = subset(deformation, select = -c(source_z,target_z,dz) )

ResultsSummary <- data.frame(stringr::str_split_fixed(files, "/",n = 6))

ResultsSummary <- mutate(ResultsSummary, deformation_sum_magnitude = 0)

ResultsSummary <- mutate(ResultsSummary, deformation_mean=0)

ResultsSummary <- mutate(ResultsSummary, Perimeter_source = 0)

ResultsSummary <- mutate(ResultsSummary, deformation_mean_divided_by_perimeter_source =0)

for (i in 1:(length(file_numlines)))

{

ResultsSummary$Perimeter_source[i]=perimeter_source[i]

ResultsSummary$deformation_sum_magnitude[i]=deformation_sum_magnitude[i]

ResultsSummary$deformation_mean[i]=deformation_mean[i]

ResultsSummary$deformation_mean_divided_by_perimeter_source[i] =index_source_new[i]

}


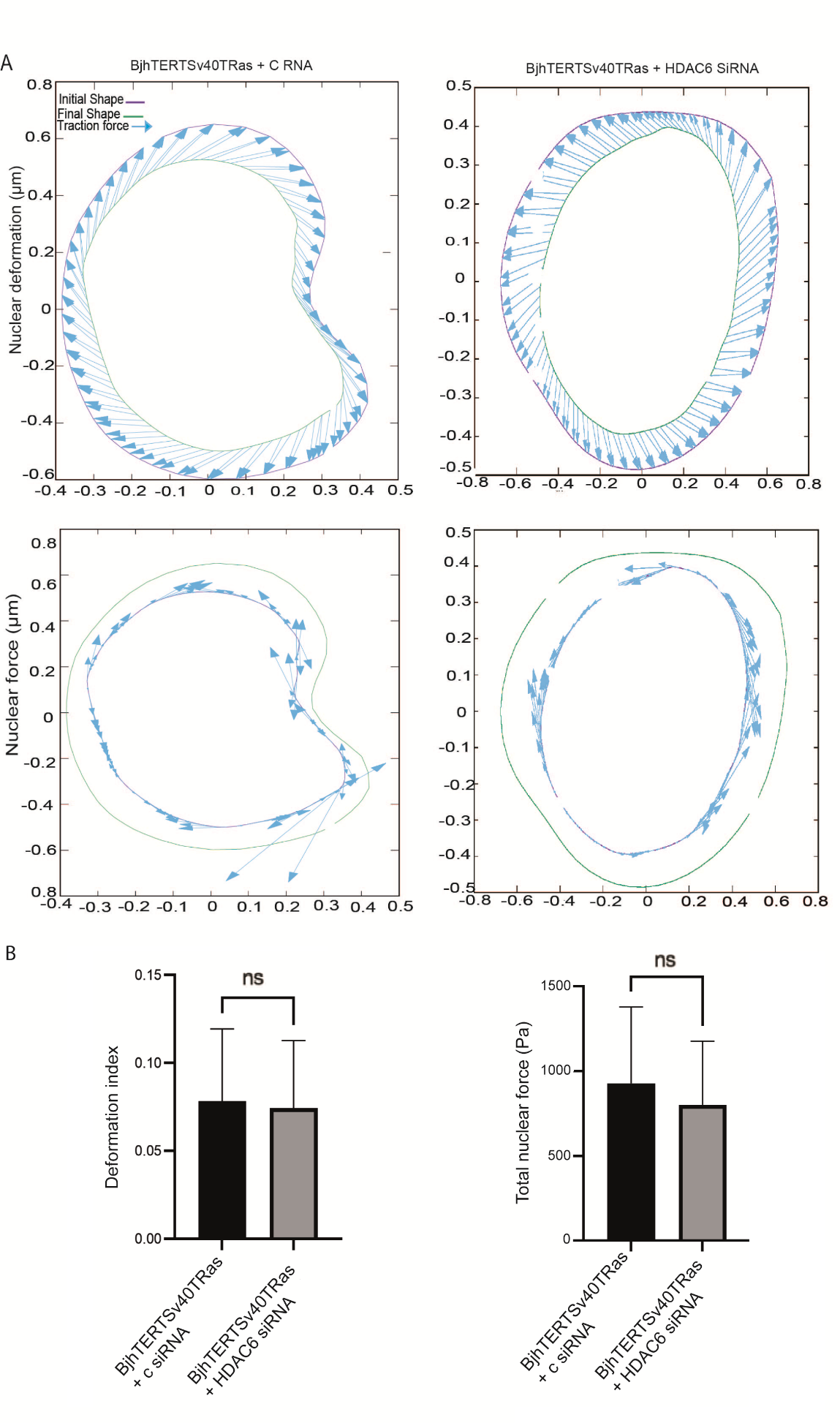


**Supplementary Figure 2. Loss of HDAC6 does not change the nuclear forces of transformed and invasive cells.** **(A)** Same cell, treated with HDAC6 siRNA or control, showing (top) the nuclear deformation, with the deformed (magenta), undeformed shape (green line) and deformation (cyan arrows), and (bottom panel) nuclear force with the undeformed (magenta), deformed shape (green line) and traction forces (cyan arrows), as indicated, and quantified in **(B)** with the deformation index (left) and total nuclear force (right). Data from at least three independent biological repeats and presented as mean ± SD. *p ≤ 0.05, **p ≤ 0.01 (t-tests).


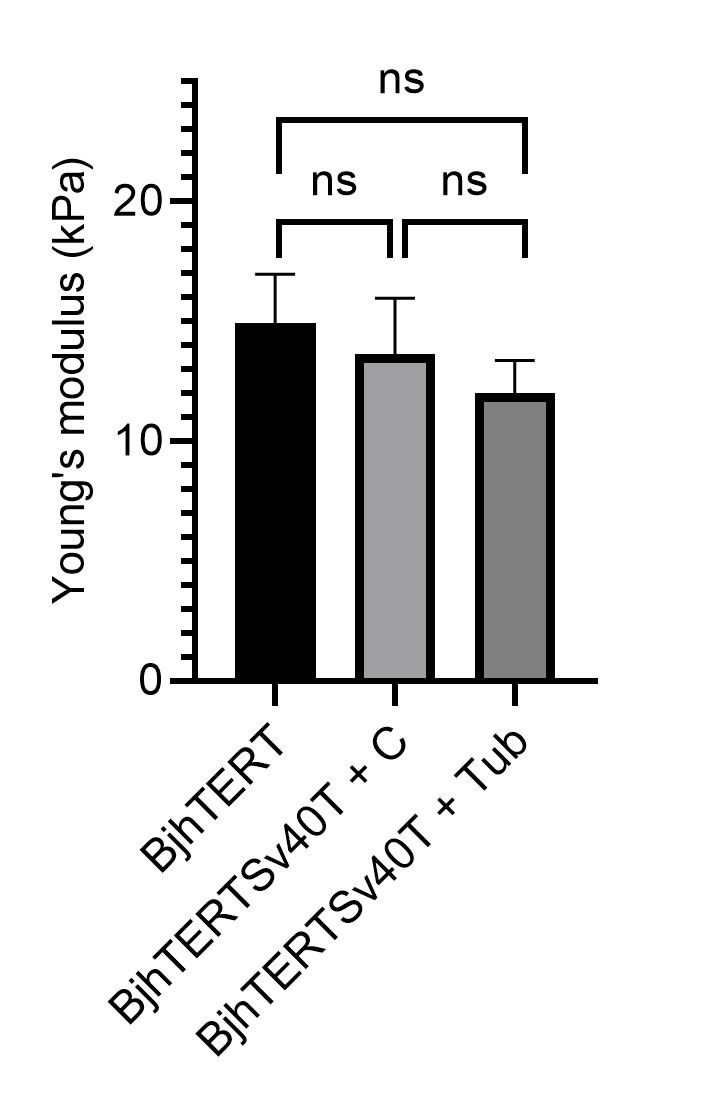


**Supplementary Figure 3. Oncogene-expression in BjhTERT cells, or HDAC6-inhibition in oncogene-expressing BjhTERT cells does not change nuclear stiffness**. Quantification of resistance to mechanical deformation (stiffness) by colloidal probe AFM indentation of cells at an applied load of 15 nN over the cell nucleus. Ten cells were analyzed for each condition. Error bars represent the SEM.


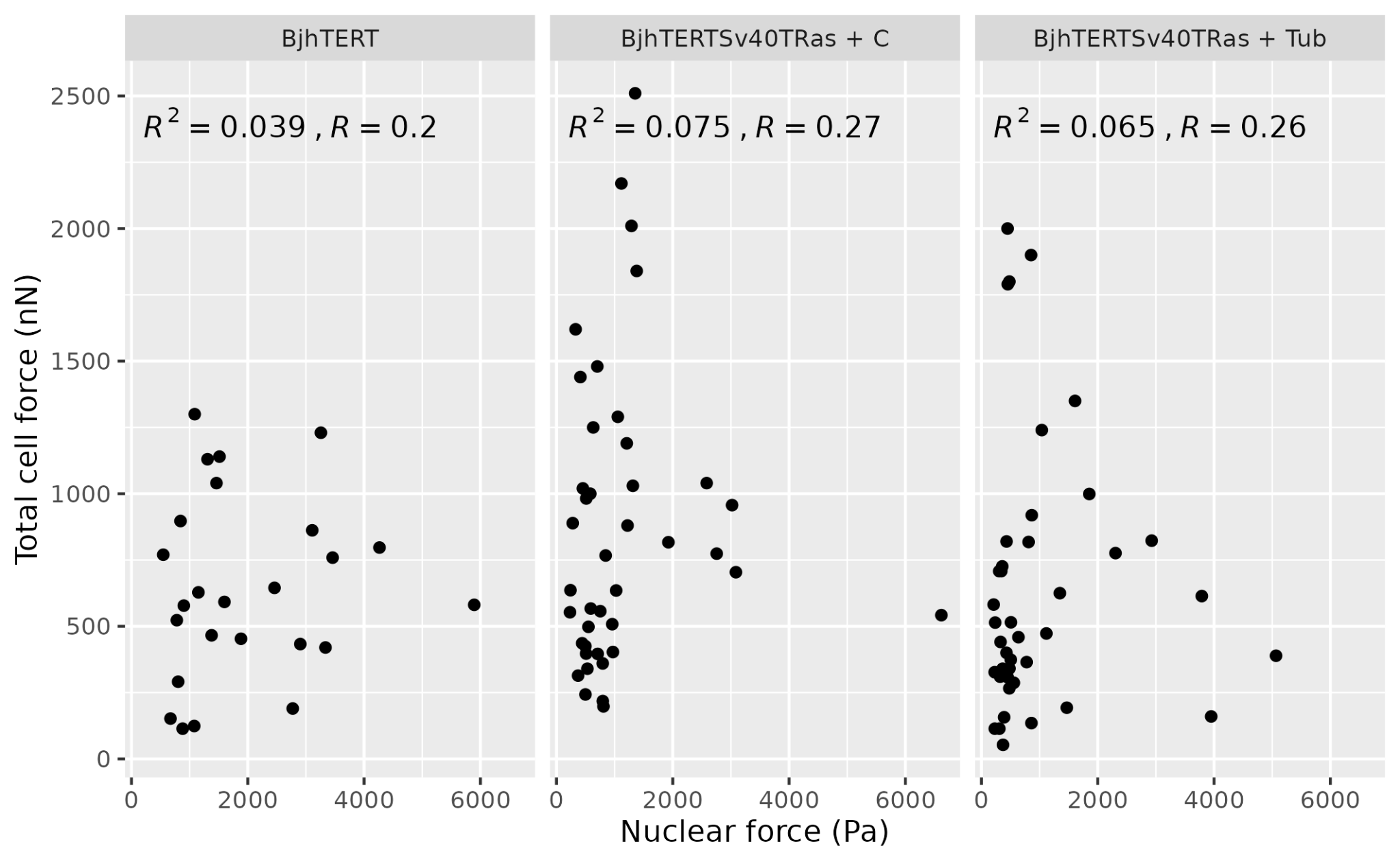


**
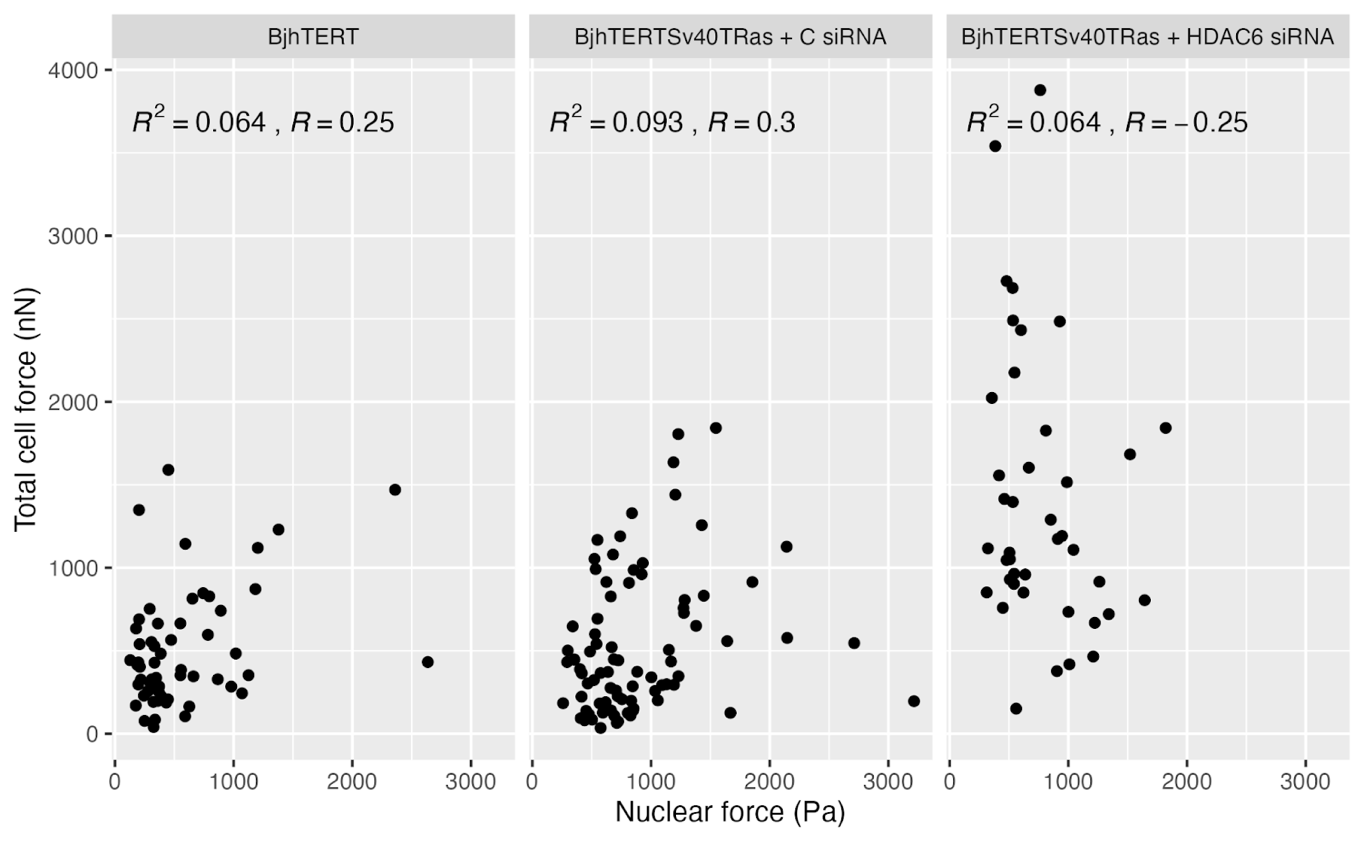
**

**Supplementary Figure 4. The Spearman correlation between total and nuclear forces is increased in oncogenically transformed and invasive cells, in a HDAC6-dependent manner.** The total force that cells exert on their surrounding environment, and the intracellular force on the nucleus for each individual, normal cells (BjhTERT), and for each oncogenically transformed and invasive cells (BjhTERTSv40TRas), treated with either **(A)** DMSO control (C) or the HDAC6-inhibitor tubacin (Tub), or (**B)** control siRNA or HDAC6-targeting siRNA, as indicated. The coefficient of determination (R^2^) and the Spearman correlation coefficient (R), were calculated and shown.


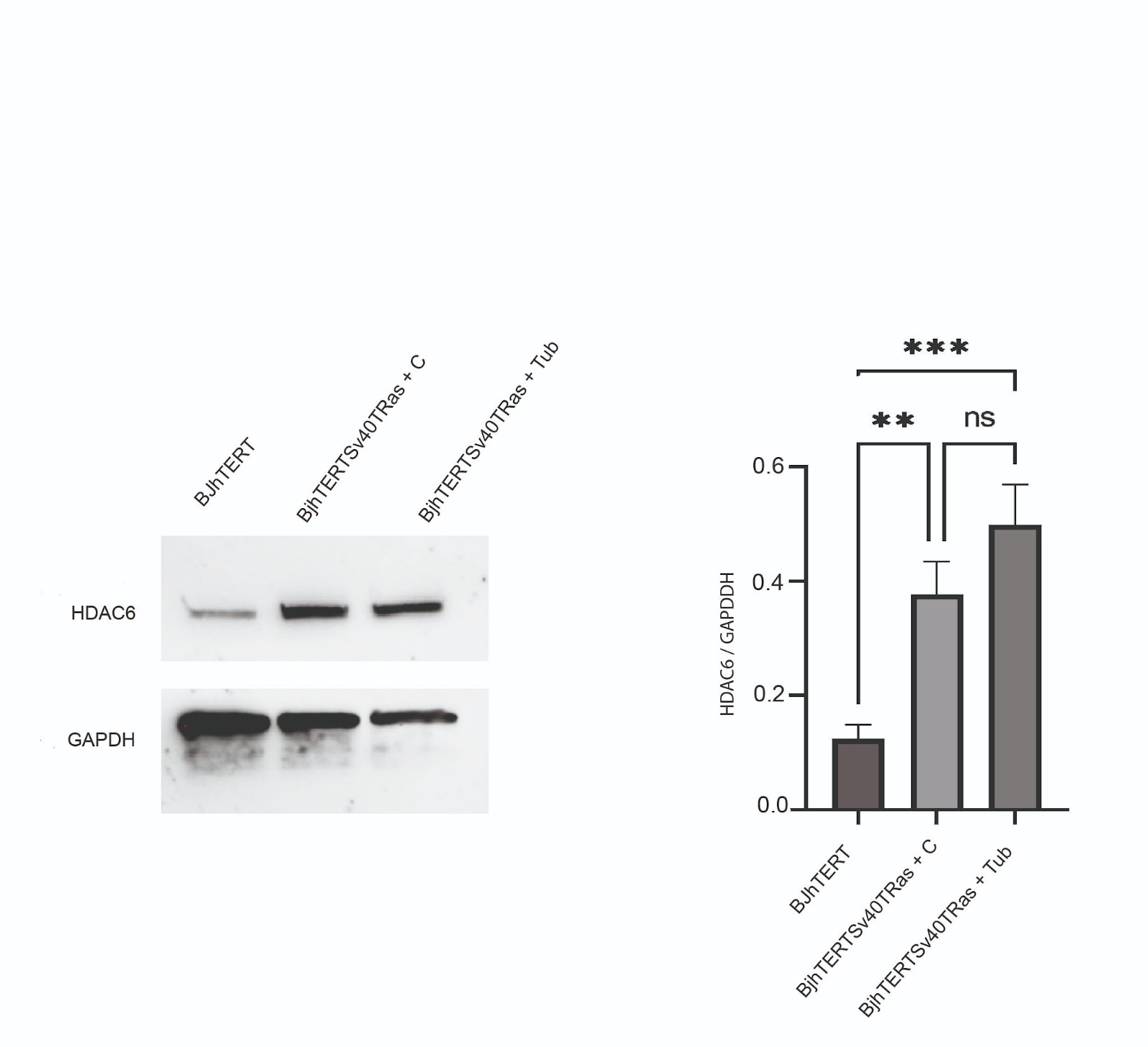


**Supplementary Figure 5. Transformed and invasive cells show increased levels of HDAC6, independent of Tubacin-treatment.** Normal BjhTERT and transformed and invasive BjhTERTSV40TRas cells treated with Tubacin (Tub) or DMSO control (C), (left), showing HDAC6 and GAPDH loading control, as indicated, and quantified (right panel). n=3, n = three independent biological, experimental repeats.

**
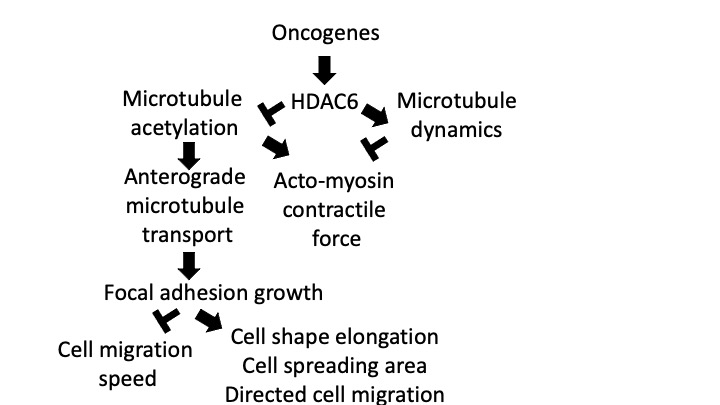
**

**Supplementary Figure 6. Summary of HDAC6-mediated control of cell forces and motility.** Hypothetical model of oncogene-induced, HDAC6-mediated control of microtubule acetylation and dynamics, cellular contractile force, cell adhesions, shape and migration.

**
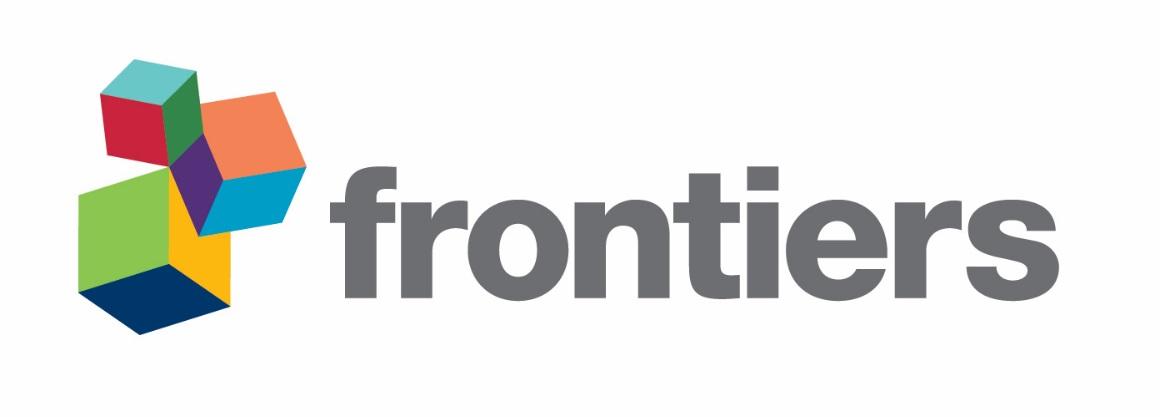
**
